# Uracil Accumulation and Mutagenesis Dominated by Cytosine Deamination in CpG Dinucleotides in Mice Lacking UNG and SMUG1

**DOI:** 10.1038/s41598-017-07314-5

**Published:** 2017-08-03

**Authors:** Lene Alsøe, Antonio Sarno, Sergio Carracedo, Diana Domanska, Felix Dingler, Lisa Lirussi, Tanima SenGupta, Nuriye Basdag Tekin, Laure Jobert, Ludmil B. Alexandrov, Anastasia Galashevskaya, Cristina Rada, Geir Kjetil Sandve, Torbjørn Rognes, Hans E. Krokan, Hilde Nilsen

**Affiliations:** 1 Department of Clinical Molecular Biology, Ahus Campus, University of Oslo, Oslo, Norway; 2 Akershus University Hospital, Lørenskog, Norway; 3 Department of Cancer Research and Molecular Medicine, Norwegian University of Science and Technology, Trondheim, Norway; 4 The Liaison Committee for Education, Research and Innovation in Central Norway, Trondheim, Norway; 5 Department of Informatics, University of Oslo, PO Box 1080 Blindern, NO-0316 Oslo, Norway; 6 MRC Laboratory of Molecular Biology, Cambridge, UK; 7 Theoretical Biology and Biophysics (T-6), Los Alamos National Laboratory, Los Alamos, NM 87545 USA; 8 Center for Nonlinear Studies, Los Alamos National Laboratory, Los Alamos, NM 87545 USA; 9 University of New Mexico Comprehensive Cancer Center, Albuquerque, NM 87102 USA; 10 Department of Microbiology, Oslo University Hospital, Rikshospitalet, PO Box 4950 Nydalen, NO-0424 Oslo, Norway; 11 LifeTechnologies AS, Ullernschauseen 52, 0379 Oslo, Norway

## Abstract

Both a DNA lesion and an intermediate for antibody maturation, uracil is primarily processed by base excision repair (BER), either initiated by uracil-DNA glycosylase (UNG) or by single-strand selective monofunctional uracil DNA glycosylase (SMUG1). The relative *in vivo* contributions of each glycosylase remain elusive. To assess the impact of SMUG1 deficiency, we measured uracil and 5-hydroxymethyluracil, another SMUG1 substrate, in *Smug1* ^−/−^ mice. We found that 5-hydroxymethyluracil accumulated in *Smug1* ^−/−^ tissues and correlated with 5-hydroxymethylcytosine levels. The highest increase was found in brain, which contained about 26-fold higher genomic 5-hydroxymethyluracil levels than the wild type. *Smug1* ^−/−^ mice did not accumulate uracil in their genome and *Ung* ^−/−^ mice showed slightly elevated uracil levels. Contrastingly, *Ung* ^−/−^ *Smug1* ^−/−^ mice showed a synergistic increase in uracil levels with up to 25-fold higher uracil levels than wild type. Whole genome sequencing of UNG/SMUG1-deficient tumours revealed that combined UNG and SMUG1 deficiency leads to the accumulation of mutations, primarily C to T transitions within CpG sequences. This unexpected sequence bias suggests that CpG dinucleotides are intrinsically more mutation prone. In conclusion, we showed that SMUG1 efficiently prevent genomic uracil accumulation, even in the presence of UNG, and identified mutational signatures associated with combined UNG and SMUG1 deficiency.

## Introduction

DNA glycosylases recognize subtle chemical modifications of DNA bases. After identification of a modification, glycosylases also excise the modified DNA base to allow an unmodified base to be inserted in its place through a series of finely-tuned catalytic steps comprising the base excision repair (BER) pathway. Uracil is one of the most prevalent non-canonical bases in DNA^1^. It is a natural intermediate in thymidine biosynthesis and deoxyuridine triphosphate (dUTP) misincorporation by replicative polymerases is a major source of genomic uracil in proliferating cells, with an estimated ~10^4^ dUTPs misincorporated per genome per cell-division cycle^1, 2^. Uracil is also generated by spontaneous and enzymatic cytosine deamination, resulting in U:G mispairs. The spontaneous cytosine deamination rate is estimated to be 100 to 500 cytosines per cell per day^1^. The mutagenic potential of genomic uracil depends on its origin: U:A pairs are presumably innocuous if unrepaired as uracil is read as a thymine by DNA polymerases. However, mutations may arise at U:A pairs after U excision due to translesion synthesis across abasic-sites^3^. In contrast, U:G mispairs give rise to C to T transition mutations, the most common type of mutation in cancer^4^. Indeed, the most common mutational signature in human cancers besides aging has been attributed to enzymatic cytosine deamination^5^. In order to understand how spontaneous mutations arise it is therefore necessary to decipher how uracil is repaired *in vivo*. Uracil is primarily excised by uracil-DNA glycosylases (UDGs)^6^, and the two UDGs capable of repairing both U:A and U:G from nuclear DNA are uracil-DNA glycosylase (UNG)^7^ and single-strand selective monofunctional uracil-DNA glycosylase 1 (SMUG1)^8^. *Ung* ^−/−^ mice have been shown to accumulate uracil in the genome, but show a modest (less than 2-fold) increase in spontaneous mutation frequency^9^. Biochemical measurements of UDG activity in *Ung* ^−/−^ mice showed that SMUG1 was contributing to most of the remaining UDG activity but its contribution in UNG-proficient cells was modest^9^. SMUG1 was therefore suggested to serve as a backup for UNG in uracil repair^10–13^. An additive increase in spontaneous C to T mutations was, however, observed in the hypoxanthine phosphoribosyltransferase 1 (*Hprt*) gene in SMUG1-depleted *Ung* ^−/−^ MEFs, suggesting that SMUG1 contributes to uracil repair *in vivo* ^14^. To explain this apparent discrepancy, it has been suggested that the enzymes may operate in distinct regions or cellular settings guided by differences in their regulation^15, 16^ and intracellular localisation^11, 17, 18^.

In addition to uracil, SMUG1 repairs 5-hydroxymethyluracil (hmU)^19, 20^ and other DNA-pyrimidine oxidation products^20, 21^, some of which, such as 5-formyluracil, are mutagenic^22, 23^. Like uracil, the mutagenic potential of hmU depends on its origin: when present in a hmU:A base pair after direct oxidation of thymidine it is likely innocuous^24^, but hmU has been suggested to form as an intermediate of oxidative demethylation of 5-methylcytosine^25^, leading to a premutagenic hmU:G mispair. These substrates are not shared between SMUG1 and UNG, which could explain the additive mutagenic effect of suppressing SMUG1 expression in *Ung* ^−/−^ MEFs.

Thus, there are several unresolved questions regarding the distinct functions of UNG and SMUG1 in *in vivo* uracil repair and their importance for limiting spontaneous mutagenesis. Here, we measured the levels of UNG and SMUG1 substrates in genomic DNA of mice deficient in one or both enzymes to clarify their relative importance in BER. We show that SMUG1 knockout mice accumulate genomic hmU. *Smug1* ^−/−^ mice did not accumulate uracil, although *Smug1* ^−/−^ brain extracts exhibited reduced uracil excision activity. Unexpectedly, *Ung* ^−/−^ *Smug1* ^−/−^ mice had more than an additive increase in uracil content compared to either single knockout. This shows that SMUG1 is important for uracil repair also in cells with functional UNG. Hence, the two enzymes complement each other during the repair of uracil *in vivo* and the mutagenic potential of defective uracil repair is best assessed in cells lacking both enzymes: whole genome sequencing of UNG/SMUG1-deficient tumours revealed that the loss of uracil repair by UNG and SMUG1 primarily leads to C to T transition mutagenesis at CpG dinucleotides.

## Results

### *Smug1*^−/−^ mice accumulate hmU in genomic DNA

**Figure 1:**
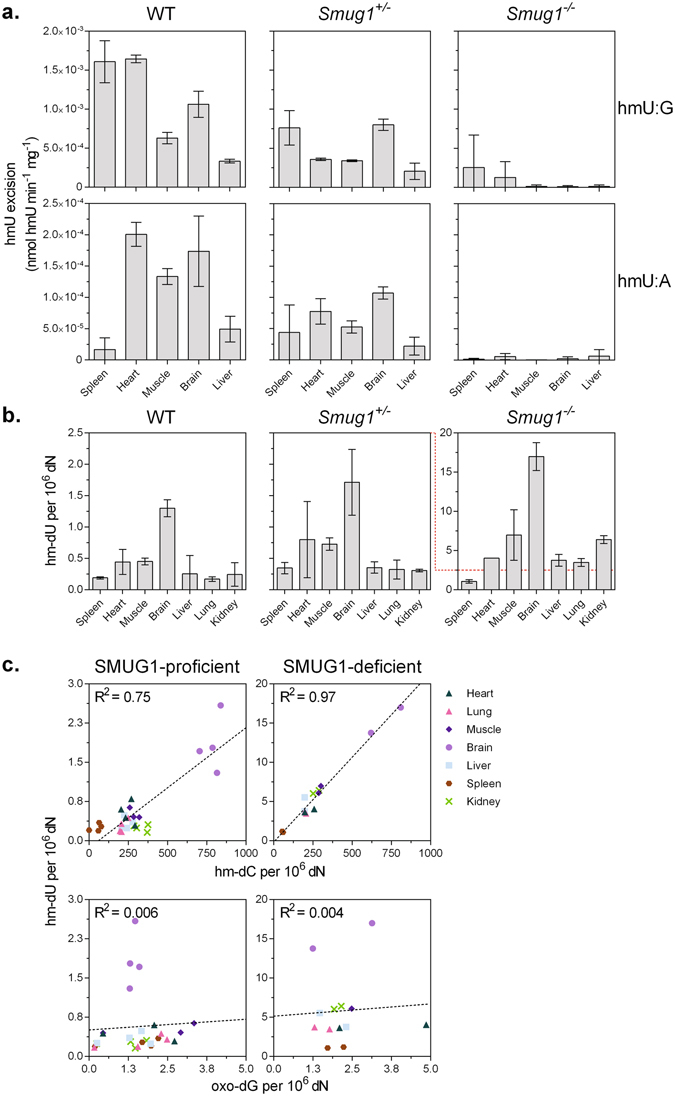
SMUG1 is responsible for hmU-excision activity in mouse tissues. (**a**) hmU excision activity measured in oligonucleotides containing a single hmU:G (top panel) and hmU:A (lower panel) base pair. Heterozygous SMUG1 knockout reduces, whereas homozygous knockout abolishes hmU excision activity. Biochemical assays were evaluated using two-tailed t-test 95% confidence level. (**b**) hm-dU levels (presented as the number of hm-dU residues per million nucleosides) increases in DNA from homozygous but not heterozygous SMUG1 knockout organs. Error bars indicate SD of three biological replicates. (**c**) hm-dU and hm-dC levels (upper panels) correlate strongly in the SMUG1-proficient group (R^2^ = 0.75, p < 0.0001) and very strongly in the SMUG1-deficient group (R^2^ = 0.97, p < 0.0001), whereas hm-dU and oxo-dG levels (lower panels) do not correlate. The effect of SMUG1 and UNG knockout on genomic lesion levels were assessed using an unpaired t-test or analysis of variance (ANOVA).

A SMUG1-deficient mouse strain (*Smug1* ^*tm1a*(*EUCOMM*)*Hmgu*−/−^) was previously generated by germline ablation of SMUG1 expression using a gene-trap^12^. Here we generated an additional allele, a classical gene targeted SMUG1-knockout mouse line (*Smug1* ^*tm1lHn*^ allele, referred to as *Smug1* ^−/−^), in which both coding exons were deleted (Supplementary Figure S1). SMUG1 was previously shown to be the main enzyme removing hmU from DNA^12, 19^. Consistently, *Smug1* ^−/−^ mice had virtually no detectable residual hmU-excision activity (Fig. 1a), when measured on an oligonucleotide substrate harbouring a centrally placed hmU residue regardless of substrate, tissue type, DNA single- or double-strandedness (data on single-stranded hmU excision not shown). Hence, phenotypic and biochemical characterisation revealed no biochemical or phenotypic differences provided by the *Smug1* ^*tm1a*(*EUCOMM*)*Hmgu*−/−12^ and the *Smug1* ^*tm1lHn*^ alleles.

In wild type mice, there were pronounced differences in the hmU-excision capacity between different tissues. The hmU excision activities were generally higher on hmU:G than on hmU:A substrates: heart, muscle, brain, and liver extracts showed modest 4.7- to 8.2-fold increases, whereas splenic extracts showed a more pronounced 95.5-fold increased activity on hmU:G compared to hmU:A substrates (Fig. 1a). In *Smug1* ^+/−^ extracts, hmU-excision activity levels were lower than WT, with a reduction of activity in the order of 1.3- to 4.6-fold on hmU:G substrate and 1.6- to 2.6-fold on hmU:A substrate. The splenic extracts were the exception and showed no statistically significant difference in hmU:A excision activity in *Smug1* ^+/−^ mice compared to WT.

Whether the lack of hmU excision activity in *Smug1* ^−/−^ extracts resulted in an increased genomic hmU load was not known: to answer this, we measured the amount of 5-hydroxymethyl-2′-deoxyuridine (hm-dU) in total genomic DNA. hm-dU levels in wild type mice ranged from 0.17 to 1.30 hm-dU per 10^6^ dN in tissues (Fig. 1b). Interestingly, brain extracts contained on average 4.5-fold higher hm-dU levels than the other tissues tested. DNA from *Smug1* ^+/−^ organs contained very similar hm-dU levels as WT (R^2^ = 0.80), suggesting that a single expressed copy of *Smug1* is sufficient to maintain basal hm-dU levels. DNA from *Smug1* ^−/−^ organs contained 5.6- to 26.3-fold more hm-dU than WT. Hence, the absence of SMUG1 leads to accumulation of genomic hm-dU most prominently in tissues with lower cellular turnover, such as the brain.

### Genomic hmU and hmC levels correlate

There is at present no method to determine the source of the genomic hmU accumulating in *Smug1* ^−/−^ tissues: thymine oxidation/hydroxylation or 5-hydroxymethylcytosine (hmC) deamination^26^. However, if hmU originated from direct thymine oxidation, a correlation with other genomic oxidation markers might be expected. To assess this, we measured the DNA oxidation biomarker 8-oxo-2′-deoxyguanosine (oxo-dG) and 5-hydroxymethyl-2′-deoxycytidine (hm-dC) concurrently with hm-dU (Fig. 1c). For organ DNA, samples were divided into SMUG1-proficient and -deficient groups because the latter (including *Smug1* ^−/−^ and *Ung* ^−/−^ *Smug1* ^−/−^) contained >5-fold more hm-dU than the former. We observed a strong correlation between hm-dU and hm-dC in the SMUG1-proficient group (R^2^ = 0.75) and a very strong correlation in the SMUG1-deficient group (R^2^ = 0.97). Neither group exhibited a correlation between oxo-dG and hm-dU. Taken together, these data suggest that genomic hm-dU may arise from hm-dC deamination in mouse tissues.

### *Smug1*^−/−^ mice have normal uracil excision activity in most tissues and do not accumulate genomic uracil

**Figure 2:**
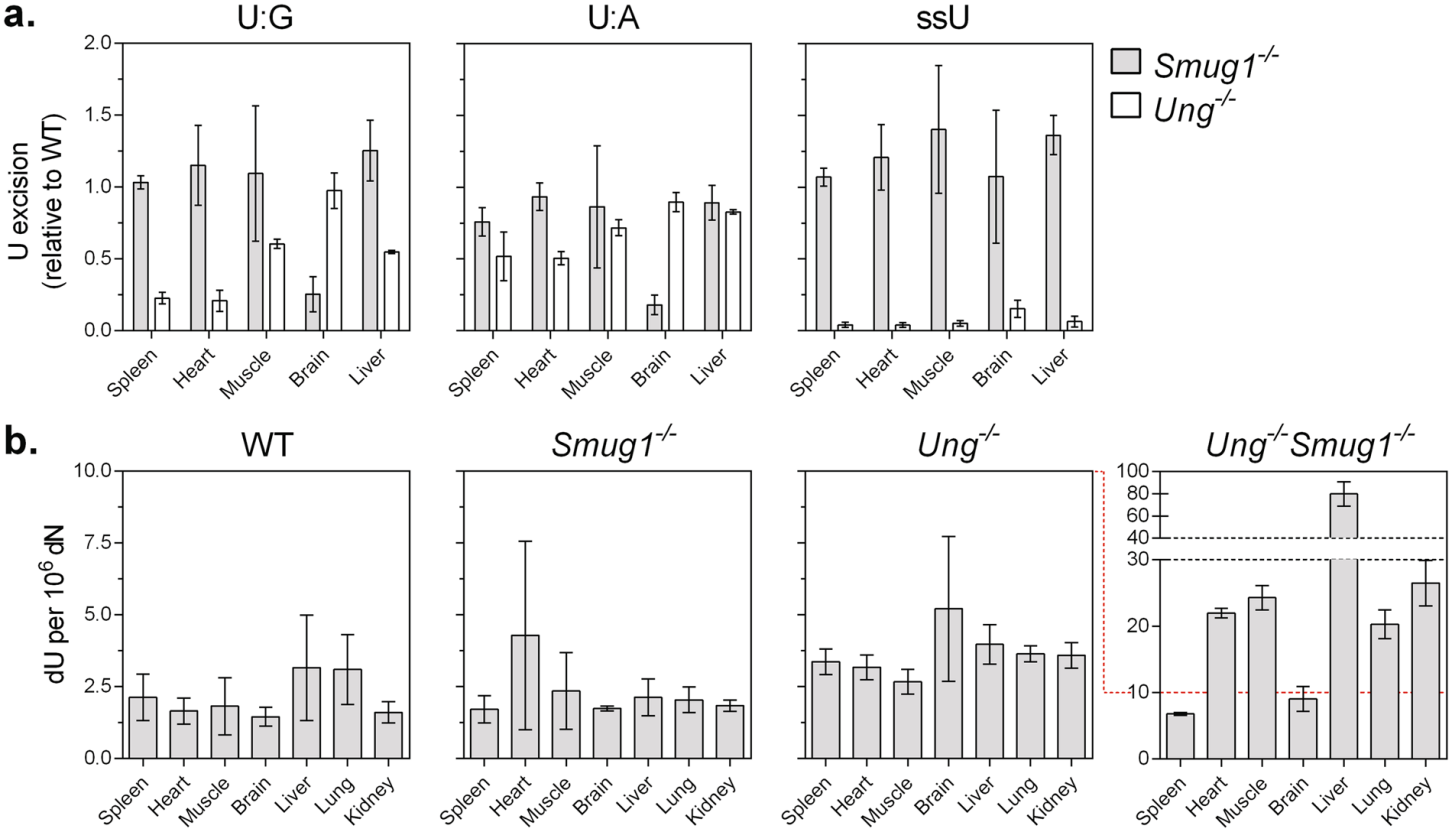
UNG and SMUG1 complement each other in U excision and UNG/SMUG1 double knockout mice accumulate a large amount of genomic uracil. (**a**) Uracil excision activity of *Ung* ^−/−^ and *Smug1* ^−/−^ organ extracts relative to WT in U:G, U:A, and single-stranded oligonucleotides. Biochemical assays were evaluated using two-tailed t-test 95% confidence level. (**b**) There was no significant genomic dU accumulation in organs from either *Smug1* ^+/−^ or *Smug1* ^−/−^ mice. Genomic dU increased slightly in *Ung* ^−/−^ and drastically in *Ung* ^−/−^ *Smug1* ^−/−^ mouse organs, respectively. The effect of SMUG1 and UNG knockout on genomic lesion levels were assessed using an unpaired t-test or analysis of variance (ANOVA). Error bars indicate SD of three biological replicates.

SMUG1 was previously found to be the predominant UDG in *Ung* ^−/−^ mice^10, 12, 27^. To assess whether the loss of SMUG1 impacted uracil repair capacity, we measured uracil excision activity on oligonucleotide substrates containing one centrally placed uracil. Similarly to hmU, and in agreement with previous reports^11^, ssU excision activity was generally highest, followed by U:G then U:A activities. In WT extracts, U excision activities varied more between organs than hmU excision activities (Supplementary Figure S3). Notably, U excision activities in spleen and heart extracts were 6.9- to 11.5-fold higher on U:G and ssU substrates, respectively, than muscle, brain, and liver extracts. U excision activity correlated very strongly between U:G and ssU substrates (R^2^ = 0.99, p = 0.0003), but not between U:A and U:G or ssU substrates (Supplementary Figure S3). Neither *Smug1* ^+/−^ nor *Smug1* ^−/−^ extracts exhibited any reduction in U excision compared to WT with the exception of brain extracts, which showed 1.4- and 3.9-fold reductions in U:G excision and 1.4- and 5.5-fold reductions in U:A excision compared to WT in *Smug1* ^+/−^ and *Smug1* ^−/−^ extracts, respectively (Fig. 2a). These results indicate that SMUG1 contributes more to overall uracil excision capacity in brain tissue than in the other organs tested^12^. Expectedly, SMUG1 did not affect ssU excision activity (Supplementary Figure S3). In agreement with U excision activity, no statistically significant accumulation of genomic 2′-deoxyuridine (dU) was seen in *Smug1* ^−/−^ DNA from any of the SMUG1-deficient organs tested (Fig. 2b). These data are in agreement with previous findings that UNG contributes most of the UDG activity in mouse cells, although here we also show that SMUG1 contributes significantly to uracil excision from double-stranded substrates in the brain.

### The combined action of SMUG1 and UNG prevents genomic uracil accumulation

To test whether SMUG1 activity contributes to uracil repair in the absence of UNG, we generated *Ung* ^−/−^ *Smug1* ^−/−^ double knockout (DKO) mice and measured U excision activity and dU levels in protein and DNA extracts from *Ung* ^−/−^ *Smug1* ^−/−^ mice. There was a complete ablation of measurable U excision activity in all *Ung* ^−/−^ *Smug1* ^−/−^ organs, regardless of substrate (Supplementary Figure S3). In most tissues, *Ung* ^−/−^ extracts exhibited 1.6- to 4.8-fold and 1.2- to 2.0-fold reductions in U:G and U:A excision activities compared to WT, respectively (Fig. 2a). U excision activity in brain extracts remained unchanged on both U:G and U:A substrates, further corroborating that SMUG1 is a prominent UDG in mouse brain tissue. Expectedly, *Ung* ^−/−^ extracts showed no UDG activity on the ssU substrate. These data confirm that UNG and SMUG1 account for the majority of U excision activity in mice, with UNG as the larger contributor.

The levels of genomic dU measured in *Smug1* ^−/−^ organs were indistinguishable from WT (Fig. 2b). UNG knockout modestly increased genomic dU levels in some of the tissues tested (Fig. 2b). We observed statistically significant increases compared to WT in DNA from *Ung* ^−/−^ heart (1.9-fold, adjusted p-value = 0.014) and kidney (2.2-fold, adjusted p-value = 0.0040). dU levels in DNA from the other tissue types tested were elevated as well, but the differences were not statistically significant.

In contrast to the relatively modest increase in *Ung* ^−/−^ tissues, there was a dramatic increase in genomic uracil levels in *Ung* ^−/−^ *Smug1* ^−/−^ organs (Fig. 2b). The spleen showed a modest 3.2-fold increase in *Ung* ^−/−^ *Smug1* ^−/−^ DNA compared to WT (from 2.1 to 6.8 dU per 10^6^ dN), whereas a 6.2-fold uracil accumulation was seen in the essentially post-mitotic brain extracts (from 1.5 to 9.0 dU per 10^6^ dN). DNA from heart, muscle, kidney, and lung all showed between 20 and 30 dU per 10^6^ dN, which represent more than 10-fold increases over WT. Finally, DNA from *Ung* ^−/−^ *Smug1* ^−/−^ livers contained 80 dU per 10^6^ dN, a 25-fold increase over WT. In contrast, DNA from *Ung* ^−/−^ *Smug1* ^−/−^ organs showed the same hm-dU levels as *Smug1* ^−/−^ tissues (R^2^ = 0.95), which was expected, as hm-dU is not a substrate for UNG. Thus, the synergistic increase in genomic uracil levels upon knockout of both UNG and SMUG1 demonstrates that the enzymes do not function in restricted regions only, as SMUG1 efficiently, albeit not fully, compensates for loss of UNG activity and *vice versa*.

### *In vivo* mutation accumulation in UNG/SMUG1-deficient tumours

**Figure 3:**
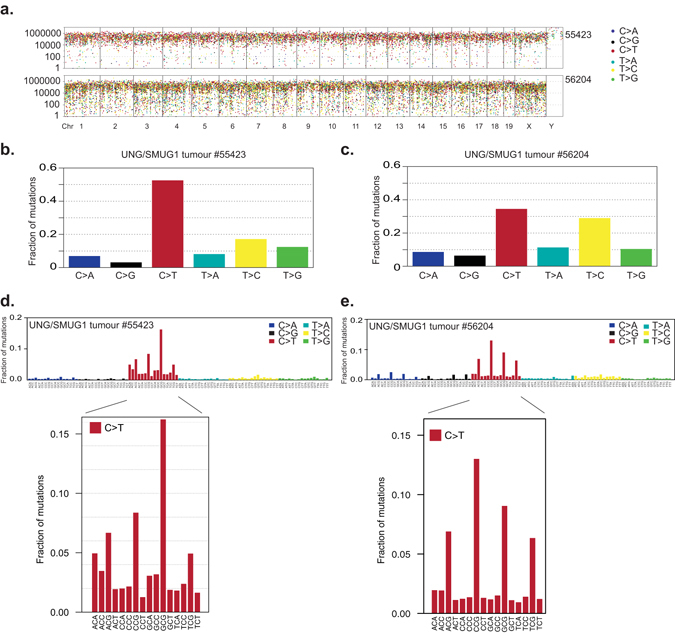
Mutational landscape of UNG/SMUG1 double knockout tumour. Whole genome sequencing was performed to assess the mutational profiles in tumour arising spontaneously in UNG/SMUG1 double knockout mice. (**a**) Rainfall plots showing the distribution of mutations in tumours from two different mice (denoted 55423 or 56201) along each chromosome. Each dot represents one variant and the distance between mutations are indicated. (**b**,**c**) The types of base pair changes represented as the fraction of the total variant observed. (**d**,**e**) Trinucleotide plots summarising the sequences surrounding each variant normalised to genomic trinucleotide occurrence. The sequence context of all mutations occurring at C:G base pairs is magnified below.

U and hmU share the property that their mutagenic potentials depend on how they are introduced: uracil arising from misincorporation during DNA replication is non-mutagenic, whereas uracil generated by cytosine deamination is mutagenic, giving rise to C to T transition mutations. As UNG and SMUG1 effectively compensate for the loss of the other with respect to *in vivo* uracil repair, the full mutagenic potential of genomic uracil will only become evident in cells lacking both enzymes. Although uracil levels rose dramatically in the *Ung* ^−/−^ *Smug1* ^−/−^ mice, they were not tumour prone, at least up to the age of 12 months in specific pathogen-free (SPF) housing. However, two cases of lymphoid tumours (more than 80% clonal based on TCR rearrangements (Supplementary Figure S4)) were previously found in a cohort of aged *Smug1* ^*tm1a*(*EUCOMM*)*Hmgu*−/−^ *Ung* ^*tm1Tld*−/−^ mice (hereafter referred to as UNG/SMUG1-DKO) at about 2 years of age^12^. To assess the impact of the combined loss of UNG and SMUG1 activity on the spectrum of mutations generated *in vivo*, we subjected these tumours to whole genome sequencing. To filter out any germline polymorphisms, variants present in both a tumour and its matched normal tissue were removed while variants found exclusively in the tumour were considered *bona fide* somatic mutations (Supplementary Table S1). Both tumours showed a relatively uniform distribution of mutations along the chromosomes (Fig. 3a) although one tumour exhibited almost 2-fold higher mutational load than the other (Supplementary Table S1). Both tumours had many closely-spaced mutations separated by fewer than 100 bp, but neither exhibited the localized hypermutation (*kataegis*) believed to be caused by local enzymatic deamination by the APOBEC family of DNA cytosine deaminases^28^. Consistently, there were no indications of enrichment of mutations in sequences that are preferential substrates for members of the APOBEC-family cytidine deaminases in any of the tumours (Supplementary Table S2). The most prevalent mutation in both tumours were C > T transitions (Fig. 3b and c). Other mutations were also identified and one tumour in particular had many T > C mutations, that could be a result of direct oxidation of thymine or may arise after the replication of substrates containing 5-hydroxymethyl- or 5-formyl- uracil (Fig. 3c). After normalization by the frequency of the given trinucleotide in the genome, however, C > T transitions emerged as the dominating class of mutation in UNG/SMUG1-DKO tumours (Fig. 3d and e). The trinucleotide plots revealed a striking non-uniform pattern where the C > T mutations occurred predominantly in NpCpG trinucleotides (mutated base is underlined) in the UNG/SMUG1*-*DKO tumours (Fig. 3d and e, respectively). A similar overrepresentation of mutations in NpCpG contexts was observed also for C > A and C > G mutations but the trinucleotide context around mutations occurring at A:T pairs were more uniform (Supplementary Figure S5).

**Figure 4:**
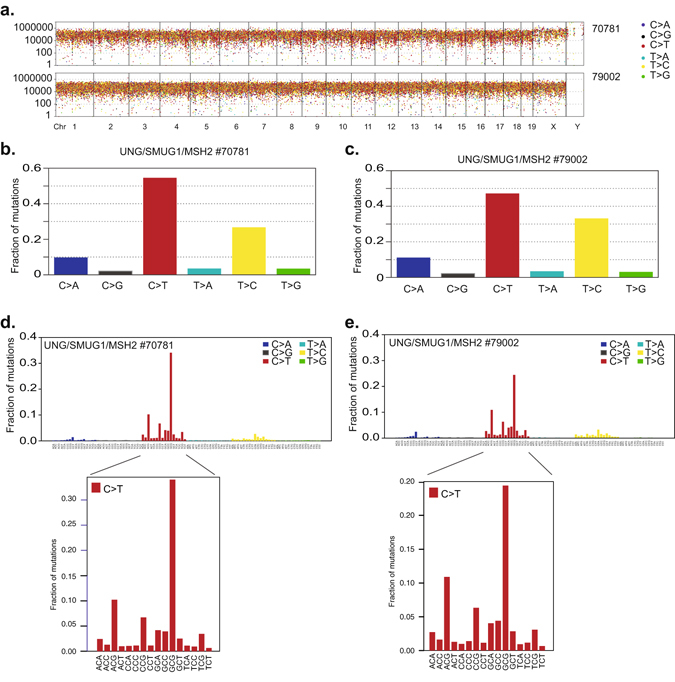
Mutational landscape of UNG/SMUG1/MSH2 triple knockout tumour. Whole genome sequencing was performed to assess the mutational profiles in tumour arising spontaneously in UNG/SMUG1/MSH2 triple knockout mice. (**a**) Rainfall plots showing the distribution of mutations in tumours from two different mice (denoted 70781 or 79002) along each chromosome. (**b**,**c**) The types of base pair changes represented as the fraction of the total variant observed. (**d**,**e**) Trinucleotide plots summarising the sequences surrounding each variant normalised to genomic trinucleotide occurrence. The sequence context of all mutations occurring at C:G base pairs is magnified below.

U:G mismatches may also be repaired by mismatch repair (MMR) and mice deficient in UNG, SMUG1, and the DNA mismatch repair protein MSH2 develop tumours much earlier (at around 4–6 months of age) and at a much higher frequency than UNG/SMUG1-DKO mice^12^. Consistently, we found that UNG/SMUG1/MSH2-triple knockout tumours have a much higher mutation load than UNG/SMUG1*-*DKO tumours (Fig. 4a and Supplementary Table S1). Both tumours analysed had a dramatic increase in C > T transitions, which constituted about half of all mutations (Fig. 4b and c). After correction for trinucleotide frequency, we see that loss of MMR resulted in a general enrichment for C > T transitions in CpG contexts (Fig. 3d and e), but with a more pronounced skewing towards mutations at GpCpG trinucleotides compared to the UNG/SMUG1*-*DKO tumours (Fig. 3d and e). The other ubiquitous class of mutations in both genotypes were T > C transitions, which together with C > T transitions constituted approximately 80% of all somatic mutations in UNG/SMUG1/MSH2-triple knockout tumours (Fig. 4b). Interestingly, C > A and C > G mutations did not retain the NpCpG context bias as was observed in the UNG/SMUG1-DKO tumours, but was instead occurring mostly in NpCpT contexts (Supplementary Figure S5). Moreover, there was a reduction in the fraction of transversions at A:T base pairs in the UNG/SMUG1/MSH2-triple knockout compared to UNG/SMUG1-DKO tumours (Supplementary Table S1).

### Mutation distribution and signatures in UNG/SMUG1-deficient tumours

**Figure 5:**
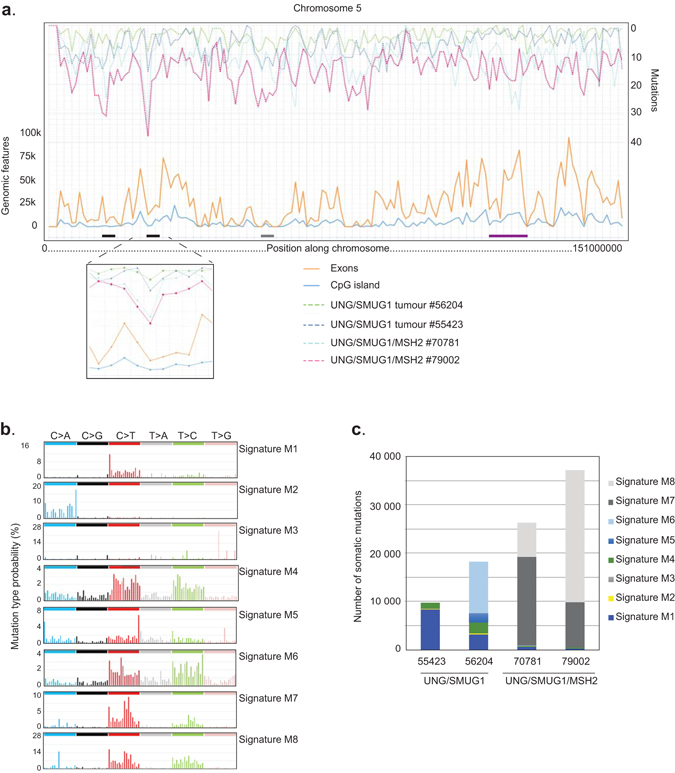
Mutational signatures. (**a**) Plot showing the mutation density (no. mutation/Mbp) along chromosome 5 for all four tumours together with the density of exons (orange) and CpG islands (blue). The 22–30 Mbp region with highest number of mutations is shown below. (**b**) Mutational signatures identified in the UNG/SMUG1 and UNG/SMUG1/MSH2 tumours. (**c**) The different mutational signatures present in each tumour given as the number of mutations contributed to each signature.

The overrepresentation of C > T transitions at CpG dinucleotides in UNG/SMUG1-DKO tumours was surprising as these are generally interpreted as resulting from 5-methylcytosine, which deamination product, thymidine, is not recognised by either SMUG1 or UNG. Therefore, to analyse whether mutations preferentially occurred in CpG islands, we divided the chromosome 5 into 1 Mbp regions and plotted the mutations along each chromosome together with genomic features extracted from the USCS Genome Browser. The mutation tracks of the two UNG/SMUG1-DKO follow each other indicating that genotype specific effects are reflected in the mutation distribution. Some regions were highly mutated in both UNG/SMUG1-DKO as well as in the UNG/SMUG1/MSH2-triple knockout tumours, but there was no general trend that mutations occurred preferentially in regions with a high density of CpG islands. Some regions, such as between 115–127 Mbp on Chromosome 5 (Fig. 5a, purple region), were highly mutated in the MMR defective tumours, but had very low mutation loads in the UNG/SMUG1 tumours. These regions tended to be gene-rich, illustrated with a high density of exons (Fig. 5a). The highest number of mutations in the triple-knockout tumours was found in two regions with low density of CpG islands that flanked exon-rich region (Fig. 5a, black regions). Regions containing many mutations in the UNG/SMUG1-DKO tumours tended to have fewer exons, for example the region between 56–60 Mbp on chromosome 5 (Fig. 5a, grey region), perhaps indicating that BER has a global genome repair function.

Next, we analysed the data with respect to mutational signatures^5^: six distinct mutational signatures, designated M1 through M6, could be deciphered from the UNG/SMUG1-DKO genomics data (Fig. 5b). Signatures M1, M2, and M4 were present in both tumours. Signature M1 resembles signature 1 from the Catalogue of Somatic Mutations in Cancer (COSMIC; http://cancer.sanger.ac.uk/cosmic/signatures), which is attributed to aging and characterised by C > T mutations (see Supplementary Figure S6 for comparisons of mouse and human signatures). Signature M2 resembles COSMIC signature 18, which has an unknown origin. Signature M4, with transitions at pyrimidines, resembles COSMIC signature 5, which also has unknown aetiology. Signature M3, characterised by CpTpT and some other trinucleotides with thymine at the 3′-end, was found in one cancer and resembles COSMIC signature 17. Signature M5, characterised by C > A or C > T at ApCpA and TpCpT trinucleotides, was also found only in one tumour, but its profile is different from any known mutational signatures identified in human cancer. Finally, signature M6, which bears some similarity with signature M4 and COSMIC signature 5, was highly represented in one of the tumours. The UNG/SMUG1/MSH2-triple knockout tumours exhibited mutational signatures resulting from very typical microsatellite instability similar to COSMIC signatures 6 and 26. Consistent with MSH2-deficiency conferring a strong mutator effect, the two tumours obtained from UNG/SMUG1/MSH2-triple knockouts were much more similar to each other with respect to mutational signatures than the two UNG/SMUG1-DKO tumours (Fig. 5c). The mutational patterns in UNG/SMUG1-DKO tumours were rather different from UNG/SMUG1/MSH2-triple knockout tumours, which enabled us to extract two additional mutational signatures (termed M7 and M8) associated with combined BER and MMR defects (Fig. 5b and c).

In conclusion, UNG and SMUG1 efficiently complement each other in uracil removal and limit a range of spontaneous point mutations in a genome-wide context *in vivo*, but their absence does not seem to be associated with one specific mutational signature.

## Discussion

Here, we used a genetic approach to clarify the relative importance of the UNG and SMUG1 DNA-glycosylases in BER. We measured UNG and SMUG1 substrates in genomic DNA of mice deficient in one, or both enzymes and found that SMUG1-deficiency leads to genomic hm-dU accumulation. The accumulated hm-dU correlated with hm-dC, indicating hm-dU may be formed by hm-dC deamination. Furthermore, we demonstrate that SMUG1 and UNG effectively collaborate to limit dU accumulation in genomic DNA leading to a synergistic increase in genomic uracil in *Ung* ^−/−^ *Smug1* ^−/−^ mice.

The work presented here offers novel insight into the long-standing question of the relative contribution of UNG and SMUG1 to the removal of genomic uracil *in vivo*. The loss of SMUG1 had no effect on U excision activity in the presence of UNG in most organs, supporting the conclusion that UNG can compensate for the loss of SMUG1 and is the major contributor to uracil excision activity in mice^9, 27, 29^. A significant reduction of UDG activity in *Smug1* ^−/−^ tissues was found only in brain extracts, suggesting that SMUG1 might have a more significant function in the brain. The dramatic reduction in U excision activity in all *Ung* ^−/−^ *Smug1* ^−/−^ organs confirms that SMUG1 is the major UDG in UNG-deficient mice^10, 12^. The slight increase in uracil levels in *Ung* ^−/−^ tissues is in line with previous estimation of uracil accumulation in UNG-deficient mouse tissues. DNA from *Smug1* ^−/−^ tissues did not accumulate dU, but DKO organs had dramatically increased dU levels. This synergistic increase was not expected as most previous studies indicated that SMUG1 contributed relatively little to *in vivo* uracil repair. The data presented here show that SMUG1 contributes globally to prevent uracil accumulation *in vivo*, even in the presence of UNG.

Biochemical studies suggest that TDG, NTH1, and NEIL1 may excise hmU^30, 31^. We were not able to detect appreciable hmU-excision activity in *Smug1* ^−/−^ tissues. Moreover, the reduction of hmU excision activity in heterozygous mice corresponded with reduction in SMUG1 expression. Further, we found no indication of differential regulation of transcripts encoding these enzymes in *Smug1* ^−/−^ liver, brain, mouse embryonic fibroblast cells, or in *Ung* ^−/−^ *Smug1* ^−/−^ organs (Supplementary Figure S1). Thus, our data suggest that SMUG1 contributes most to hmU–excision activity *in vivo*, which is in line with previous reports^19, 20^. Nevertheless, it is possible that the activity of other hmU DNA glycosylases may be underestimated under our assay conditions and we can not exclude the possibility that other glycosylases may have a specialized roles in hmU repair (*e*.*g*. in certain sequence contexts) but further genetic studies are required to evaluate these possibilities.

Whether hmU should be regarded, simply, as a DNA damage or whether it may have a function is still unclear. The presence of hmU has been shown to affect the binding of chromatin binding proteins^24^ and it was proposed that hmU may function as an epigenetic mark^32, 33^. Theoretically, hmU may be generated as an intermediate in TET-TDG-mediated active demethylation, but this would require enzymatic deamination of hmC by a cytidine deaminase, for example by the APOBEC enzymes. To our knowledge, no robust hmC demethylation activity has been found for APOBEC enzymes^34^. Recent work has, however, shown that hmC accumulates at DNA damage foci, and it is possible that these hmC-rich foci are more prone to deamination^35^. Here, we found that the differences in hm-dU between organs reflected the hm-dC levels. This is in line with previous reports^36^, and suggests that hm-dU may arise from hm-dC deamination in the organs tested.

Moreover, it has been shown that hmU can be derived from TET-mediated oxidation of thymine in mouse embryonic stem cells^24^. Using oxo-dG as a marker of general DNA oxidation^24^, we found no correlation between hm-dU and oxo-dG levels. In our experience, oxo-dG levels correlate with oxidative lesions (*e*.*g*. 5-hydroxy-2′-deoxycytidine, 8-oxo-2′-deoxyadenosine, and hm-dU) when cells are treated with oxidative agents (*A*.*S*. *unpublished results*). Taken together, these results argue against hmU being generated primarily by non-enzymatic oxidation of T, but it is still possible that TET-dependent T oxidation is important in distinct genomic regions or that it is a feature of stem cells. Subsequent work is required to elucidate whether hmU is truly derived from hmC deamination instead of thymine oxidation (*e*.*g*. by TET proteins). Furthermore, although we cannot show whether the deamination is spontaneous or enzymatic, the spontaneous deamination rate under physiological conditions is too low to explain the large amount of hm-dU measured^32, 37^.

As both UNG and SMUG1 efficiently remove uracil from U:A and U:G pairs, we cannot conclude whether the main increase in genomic uracil in the *Ung* ^−/−^ *Smug1* ^−/−^ mice comes from dUTP misincorporation or cytosine deamination. The rates of spontaneous cytosine deamination are expected to be largely dependent on the degree of single-stranded DNA and therefore on the transcription activity of a cell^1^ and unlikely to differ in the genetic background studied here. There are also other enzymes and pathways that can repair U:G mismatches in the absence of UNG and SMUG1, most notably the mismatch repair pathway and the two mismatch-specific uracil-DNA glycosylases TDG and MBD4^38–40^. The fact that C to T transition mutations were the dominating class of mutation found in the two UNG/SMUG1-deficient tumours showed that neither the other UDGs nor the MMR pathway are able to fully compensate for the loss of UNG and SMUG1 in U:G repair at CpG dinucleotides. There was no obvious bias toward mutation accumulation in regions with a high density of CpG islands which suggests that cytosine in CpG dinucleotides are intrinsically more mutation prone also in the unmethylated state.

Unfortunately, we cannot separate the relative roles of UNG and SMUG1 in mutation avoidance because neither of our single knockout mice developed tumours under SPF conditions. Nor we can use WGS of normal tissue to estimate the rate of mutation accumulation in UNG/SMUG1-DKO relative to wild-type mice because the somatic mutations would not be present in sufficient number of cells to be unequivocally assigned as a mutation. Thus, a deeper analysis of their individual contributions to *in vivo* mutation accumulation is better performed in suitable cell models. However, suppression of SMUG1 was previously shown to lead to a mild mutator phenotype, with about 2-fold increase in C to T mutagenesis, and acts additively with UNG in preventing C to T mutations in MEFs^14^.

For U:A the two most efficient repair enzymes are UNG and SMUG1^11^. Uracil misincorporation will occur in replicating cells in direct proportion to the cellular dUTP pool, which is largely determined by the dUTPase enzyme DUT, whose expression is cell-cycle regulated but not differentially expressed in *Smug1* ^−/−^ mice (Supplementary Figure S1). A relatively low increase in uracil content in *Ung* ^−/−^ *Smug1* ^−/−^ brain samples, in which there is very low cellular turnover, might indicate that the bulk of genomic uracil in the *Ung* ^−/−^ *Smug1* ^−/−^ organs originates from uracil misincorporation, which is determined by the relative cellular concentrations of dUTP and dTTP^41^. Few enzymes other than UNG and SMUG1 are known to effectively repair misincorporated uracil, which suggests that the dramatic increase in uracil content in the *Ung* ^−/−^ *Smug1* ^−/−^ mice is likely dominated by U:A pairs. The absence of a tumour-prone phenotype before the age of 12 months^12^ would also support this interpretation.

In conclusion, there is extensive buffering between UNG and SMUG1 with respect to global genome uracil repair *in vivo*. As demonstrated by the synergistic increase in uracil levels in the double knockout mice, their combined action effectively prevents accumulation of uracil in the mouse genome.

## Materials and Methods

### Mouse strains

The generation of *Ung* ^−/−^ (*Ung* ^*tm1Tld*^) mice in a mixed 129SV-C57Bl/6J background was described previously and backcrossed ten generations into the C57Bl/6J background^9, 27^. The gene-targeted *Smug1* ^−/−^ (*Smug1* ^*tm1Hln*^) mice were generated as described in the Supplementary materials and methods. The *Ung* ^−/−^ *Smug1* ^−/−^ double-knockout (DKO) mice were generated by crossing single-knockout mice *Ung* ^*tm1Tld*^ and *Smug1* ^*tm1Hln*^ born to heterozygous mothers. All strains were maintained as heterozygotes. These mouse strains were breed and housed at a specific pathogen-free (SPF) animal facility at the Norwegian Institute of Public Health. The average housing temperature was 23 °C and the average humidity was 50% during the experimental period. Health reports of the sentinels from the room have not shown any infection in the experimental period. The mice were fed RMI pellets (Special Diets Services) and they had access to water and food *ad libitum*. The animal experiments were approved by The Norwegian Food Safety Authority (Project no. 3597). *Smug1* ^*tm1a*(*EUCOMM*)*Hmgu*−/−^ *Ung* ^*tm1Tld*−/−^ (UNG/SMUG1) mice and the *Smug1* ^*tm1a*(*EUCOMM*)*Hmgu*−/−^ *Ung* ^*tm1Tld*−/−^ *Msh2* ^−/−^ (UNG/SMUG1/MSH2) mice were described earlier^12^. Experiments involving these mouse strains were performed under the EU directives and UK Home Office Project License 70/7571 with consent of the LMB Animal Welfare and ethical Review Body.

### Protein extraction for oligonucleotide nicking assays

From the Norwegian mouse cohorts protein was extracted from organs from three mice of each of the genotypes: wild type (WT), *Ung* ^−/−^, *Smug1* ^+/−^, *Smug1* ^−/−^, *Ung* ^−/−^ *Smug1* ^−/−^ using two buffers. Lysis buffer I contained 10 mM Tris-HCl (pH 8.0) and 200 mM KCl and lysis buffer II contained 10 mM Tris-HCl (pH 8.0), 200 mM KCl, 40% glycerol, and 0.5% NP-40 alternative. Buffers were freshly supplemented with 1 µM DTT, 1X Complete Protease Inhibitor Cocktail, and 1X Phosphatase Inhibitor Cocktails 2 and 3. All steps were performed with ice-cold buffer and on ice or at 4 °C. Protein from organs were lysed by suspending organs in 1.8 µl 1:1 lysis buffers I and II per mg organ and homogenization with Dounce homogenizers. The homogenates were then incubated for 2 h at 4 °C and centrifuged at 16,100 rcf. The supernatants were finally aliquoted to new tubes, snap frozen in liquid nitrogen, and stored at −80 °C.

### Isolation of genomic DNA for quantification of modified bases

From the Norwegian mouse cohorts genomic DNA was isolated from organs from three mice of each of the genotype: WT, *Ung* ^−/−^, *Smug1* ^+/−^, *Smug1* ^−/−^, *Ung* ^−/−^ *Smug1* ^−/−^ using the DNeasy Blood & Tissue kit according to the manufacturer’s instructions with minor modifications. Briefly, 2 ml CK14 homogenisation tubes containing ceramic 1.4 mm zirconium oxide beads were prepared with 400 µl ice-cold lysis buffer consisting of: 360 µl ATL buffer, 40 µl Proteinase K and 0.1 µg/µl RNaseA. 10–25 mg tissue per tube was homogenised at 4 °C by bead beating for 30 s 3× with 30 s pauses between cycles. The lysates were recovered from the beads through two needle holes in the lid of the homogenisation tube as the tube was spin upside down inside a 15 ml tube at 250 rcf for 2 min. The lysates were incubated in a water bath for at least 1 h at 37 °C and vortexed occasionally. The lysates were frozen overnight until further processed. After thawing each lysate was split into two spin columns. The final DNA was eluted in 200 µl milli-Q water per column.

### Oligonucleotide nicking assays

Oligonucleotide UDG assays were performed as previously described^11, 27^. 6-carboxyfluorescein-labeled uracil- or hydroxymethyluracil-containing oligonucleotides (5′-[6-FAM]-CATAAAGTG-U/hmU-AAAGCCTG) were annealed to 1.5x of the complementary strand containing G or A opposite U/hmU. Activity was measured by incubating 20 nM substrate with protein extracts in reactions containing 20 mM Tris-HCl (pH 8.0), 60 mM NaCl, and 1 mM EDTA, 1 mM DTT, and 0.5 mg/ml BSA. Different incubation times and extract amounts were used to ensure that all samples lay within the linear range of the assay (Supplementary Figure S2). Thus, the reactions were incubated at 37 °C for 60 min, 20 min, and 15 min for hmU:A, hmU:G, or sshmU substrates, respectively. Furthermore, 5 µg extract were used for all tissues on (hm)U:A substrate and muscle, brain, and liver extracts on (hm)U:G and ss(hm)U substrates; 0.625 µg extract were used for spleen and heart extracts on (hm)U:G and ss(hm)U substrates. The reactions were stopped on ice and abasic sites were cleaved by adding 10% ice-cold piperidine and subsequent incubation at 90 °C for 20 min. Next, the reactions were vacuum centrifuged at 60 °C for 1 h to dryness and redissolved in 60% formamide loading buffer containing 0.05% bromophenol blue. The substrate and product were separated by electrophoresis on a urea-PAGE gel containing 12% acrylamide:bis-acrylamide (19:1) and 42% urea in 0.5× TBE and visualized using a Typhoon Trio imager. Analysis was performed using ImageQuant 7 TL.

### Quantification of modified bases in genomic DNA

Modified bases were quantified by hydrolysing DNA to nucleosides, which were analysed by LCMSMS. Deoxyuridine (dU) analysis required an additional HPLC purification step before LCMSMS analysis, as previously described^42^. Prior to hydrolysis, RNA and free nucleotides were removed from the DNA samples by treatment with 4 µg RNase A and 0.5 mU/µl alkaline phosphatase in reactions containing 10 mM ammonium bicarbonate (pH 7.0) 10 mM MgCl_2_ for 30 min at 37 °C, followed by isopropanol precipitation. Next, DNA samples were hydrolysed and dephosphorylated to single nucleosides. For dU, up to 15 µg DNA were treated with 0.1 U Nuclease P1, 2 U DNase I in reactions containing 10 mM ammonium acetate (pH 6.0), 10 mM MgCl_2_, 1 mM CaCl_2_ for 30 min at 37 °C, followed by adding 0.1 U alkaline phosphatase and 100 mM (final) ammonium bicarbonate (pH 7.6) and incubating for 20 min at 37 °C. The samples were then precipitated with acetonitrile and the supernatants were vacuum centrifuged at room temperature until dry. To separate dU from dCyd, the samples were fractionated on an Agilent 1100 HPLC system using a mixed mode Primesep 200 column (2.1 mm × 150 mm, 5 µm, SieLC) with water and acetonitrile containing 0.1% formic acid as the mobile phase. The dU-containing fractions were vacuum centrifuged until dry, redissolved in 5% methanol and analysed for dU by LCMSMS using a reverse phase column (2.1 mm × 150 mm, 3.5 µm, Zorbax SB-C18, Agilent Technologies) on an LC-20AD HPLC (Shimadzu) coupled to an AB SCIEX 5500 triple quadrupole mass spectrometer with an electrospray ion source (AB SCIEX). The mobile phase consisted of water and methanol containing 0.1% formic acid. Analysis was performed in positive ionization multiple reaction monitoring mode, using the mass transitions 228.994 **→** 113.0 and 232.0 **→** 116.0 for dU and ^13^C^15^N_2_-dU (as an internal standard), respectively.

For the other non-canonical nucleosides, DNA hydrolysis was achieved by treatment with 0.1 U nuclease P1, 50 U Benzonase nuclease, 0.8 mU snake venom phosphodiesterase, and 0.1 U alkaline phosphatase in 25 µl reactions containing 20 mM ammonium bicarbonate (pH 7.6), 1 mM MgCl_2_, and 0.1 mM ZnCl_2_ for 1 h at 37 °C. Nucleosides were analysed by LCMSMS using the aforementioned Primesep 200 column on an LC-20AD HPLC coupled to an AB SCIEX 5500 triple quadrupole mass spectrometer with an electrospray ion source. Analysis was performed in positive ionization multiple reaction monitoring mode, using the mass transitions 259.0 **→** 143.1 for 5-hydroxymethyl-2′-deoxyuridine, 257.6 **→** 142.1 for 5-hydroxymethyl-2′-deoxycytidine, 284.0 **→** 168.1 for 8-oxo-2′-deoxyguanosine, and 289.0 **→** 173.1 for ^15^N_3_^13^C_2_-8-oxo-2′-deoxyguanosine (as an internal standard).

### Whole genome sequencing

Tumour genomic DNA was isolated from *Smug1* ^*tm1a*(*EUCOMM*)*Hmgu*−/−^ *Ung* ^*tm1Tld*−/−^ mice (male mouse 749 days old, and female 706 days old) and two *Smug1* ^*tm1a*(*EUCOMM*)*Hmgu*−/−^ *Ung* ^*tm1Tld*−/−^ *Msh2* ^−/−^ mice (UK cohorts)^12^ using the PureGene kit and assessed for clonality by screening HindIII and EcoRI digests for clonal rearrangement of the T-cell receptor by Southern Blot^43^. To generate probe TCRbC1int.2, primers FD156 and FD157 were used to amplify a 925 bp fragment between the Jb1 and Cb1 segments, and probe TCRbC2int.2 was generated using primers FD158 and FD159, yielding a 697 bp fragment between the Jb2 and Cb2 segments. The primer sequences are as follows FD156 5′-TGTGCCTGTGTTGGATGACC-3′, FD157 5′-TGGCATGGTTCCTGTCCATC-3′, FD158 5′-AATCTCCGGGAGGGAAATCG-3′, and FD159 5′-GGATCCTAAGGGGTTTCAAGCA-3′. PCR-amplified probe template fragments were gel-purified, quantified, and then 50 ng per probe was labelled in separate reactions with 1.85 MBq of H_3_PO_4_^32^P-dCTP using the NEBlot kit according to the manufacturer’s instructions or the DecaLabel DNA labelling kit using 100 ng input DNA to increase specific activity. Samples with at least ~80% clonal tumours were selected for analysis (Supplementary Figure S4): UNG/SMUG1 double knockout mice ID#55423 (male mouse, 749 days old) and ID#56204 (female, 706 days old) and UNG/SMUG1/MSH2 triple knockout mice ID#70781 (male, 103 days old) and ID#79002 (female, 152 days old).

Whole genome sequencing was performed using PCR-free libraries supplied by the CRUK Cambridge Institute Genomics Core or procured from the Kinghorn Centre of Clinical Genomics (KCCG) at the Garvan Institute of Medical Research, Sydney, Australia. Sequencing was performed to a minimum of 30X coverage using Illumina HiSeq technology with 150 bp (mouse #55423 and #56204) and 100 bp (mouse #70781 and #79002) paired-end reads. Each tumour sample was compared to control non-tumour (tail or brain samples) sample from the same animal to allow unequivocal assignment of mutations and control for germline mutations.

### Sequence analysis

The FASTQ file obtained for each sequencing lane was initially quality checked using FastQC 0.11.2^44^. If necessary, base quality encodings were converted using VSEARCH 2.0.1^45^. Further analysis was based on the Broad Institute best practices^46^. Reads were aligned to the mouse GRCm38 (mm10) reference genome sequence^47^ using BWA-MEM 0.7.10^48, 49^. Mates were then fixed and results converted to bam format and sorted using samtools 1.1^50^. Duplicates were marked using biobambam markduplicates2^51^ and indexed using samtools. Realignment around indels including known indels in dbSNP 138^52^ (obtained from EnsEMBL release 74) and subsequent base recalibration was performed using GATK 3.2.2^53, 54^. The results for all lanes were then merged and reindexed for each sample using samtools. Marking duplicates, indexing and indel realignment was performed again for each sample as a whole. Potential ambiguities due to mapping errors were removed. Mutations were scored as variants that appeared in the tumour but were absent in non-tumour tissue from the same animal. Single nucleotide variants were called using MuTect 1.1.7^55^. Mutations detected in regions of the reference genome containing simple repeats or low-complexity DNA identified by VSEARCH using the Dust method were ignored. Single nucleotide variants were classified into the six possible basepair substitutions based on the pyrimidine of the mutated base pair (C > A, C > G, C > T, T > A, T > C, and T > G) and counted. The UCSC Genome Browser^56^ and IGV 2.3.26^57^ was used for visual inspection of mapped reads and mutated sites. An inverted analysis to identify mutations in normal tissue that were not found in tumour tissues was performed to confirm that the mutations assigned was not artifacts caused by C-deamination during sample work up, library preparation and sequencing as any artefactual deamination should apply equally to all samples The inverted analysis shows a much lower number of mutations and a much more even distribution of mutation types which would not be expected if resulting from artifactual C deamination (Supplementary Table S3).

Plots of mutation frequencies and rainfall plots^58^ were generated using Gnuplot 4.6.3.

### Mutational signatures

Mutational signatures were detected by *de novo* extraction based on somatic substitutions and their immediate sequence context. More specifically, single base mutations were classified into six types: C > A, C > G, C > T, T > A, T > C, and T > G (all single base mutations are referred to by the pyrimidine of the mutated Watson–Crick base pair). This classification was further elaborated by including both the 5′ and 3′ base immediately next to the mutation, resulting in 96 possible mutation types. The *de novo* extraction was performed using a previously developed theoretical model and its corresponding computational framework^59^. Briefly, while avoiding overfitting of the data, the algorithm deciphers the minimal set of mutational signatures that optimally explains the proportion of each mutation type in each mutational catalogue and then estimates the contribution of each signature to each sample. The mutational signatures extracted from the mouse data were also re-normalized to the trinucleotide frequency of the mouse genome and compared to the mutational signatures previously found by analysing more than 12,000 human cancers (http://cancer.sanger.ac.uk/cosmic/signatures). Plots of normalized mutations frequencies in different trinucleotide sequence contexts were plotted using Galaxy and the Genomic Hyperbrowser^60^.

### Statistical analysis

The effect of SMUG1 and UNG knockout on genomic lesion levels were assessed using an unpaired t-test or analysis of variance (ANOVA) on group differences, based on three samples in each group. The resulting p-values were adjusted for multiple testing according to the Holm-Šídák procedure and reported as adjusted p-values using GraphPad software (v6.07). The level of significance was set at p < 0.05. Biochemical assays were evaluated using two-tailed t-test 95% confidence level.

## Electronic supplementary material

Supplementary Data File
