# Supplementary material for: Uracil Accumulation and Mutagenesis Dominated by Cytosine Deamination in CpG Dinucleotides in Mice Lacking UNG and SMUG1
Source: Sci Rep. 2017 Aug 3;7:7199. doi: 10.1038/s41598-017-07314-5 (PMC5543110; doi:10.1038/s41598-017-07314-5)
Supplement: Supplementary file 1 — Supplementary Data File [file 41598_2017_7314_MOESM1_ESM.pdf]

URACIL ACCUMULATION AND MUTAGENESIS DOMINATED BY CYTOSINE DEAMINATION IN C_PG DINUCLEOTIDES IN MICE LACKING UNG AND SMUG1

Lene Alsøe[1#], Antonio Sarno[2#], Sergio Carracedo[1], Diana Domanska[3], Felix Dingler[4], Lisa Lirussi[1], Tanima SenGupta[1], Nuriye Basdag Tekin[1], Laure Jobert[1,9], Ludmil B. Alexandrov[5, 6, 7], Anastasia Galashevskaya[2], Cristina Rada[4], Geir Kjetil Sandve [3], Torbjørn Rognes[3,8], Hans E. Krokan[2], Hilde Nilsen[1*]

LIST OF SUPPLEMENTARY TABLE AND FIGURE LEGENDS

Supplementary Materials and Methods
Supplementary Figure S1
Supplementary Figure S2
Supplementary Figure S3
Supplementary Figure S4
Supplementary Figure S5
Supplementary Figure S6
Supplementary Table S1. Number of mutations observed
Supplementary Table S2. Sequence contexts of mutations in mouse tumours
Supplementary Table S3. Inverted mutation analysis
Full Western blots

SUPPLEMENTARY MATERIALS AND METHODS

Chemicals and Enzymes

All buffers and enzymes were obtained from Sigma-Aldrich unless otherwise specified.

Generation of gene-targeted *Smug1*^{tm1Hln} knockout mice

The *Smug1* gene (NM_027885) spans 14 kb and eight predicted exons of which only the last two exons are protein coding. Two isoforms exist that differ in their 5'-UTRs. A two-step strategy giving a final deletion of the protein coding exons 1 and 2 was used. A gene targeting vector containing *Smug1* exon 1 and exon 2 was constructed by cloning genomic *Smug1* fragments PCR amplified from C57Bl/6 genomic DNA using Accuprime Taq DNA polymerase High Fidelity (Thermo Scientific) into the pCR4-TOPO vector (Thermo Scientific). The long-homology arm of the targeting vector was built by TA-cloning of two separate PCR fragments: i) a 3371 bp fragment comprising 5´-UTR was amplified using forward primer 5´-TAGATGTGGTGGGGATAGACTAGAACCTGG-3´ and reverse primer 5´-CTACAAGCTCACTTTCCTGGTAACGAAGG-3´ and ii) a 2464 bp fragment comprising the exon 1 and exon 2 regions amplified using the primers (5´-TGACTGACAGGGTTTCTTCTGAGCCC-3´ and 5´-GAAGGGGAAGACAGCAGGAGAGCTG-3´) flanked by two LoxP sites. A positive selection neomycin gene flanked by FRT-sites was inserted. The short homology arm was generated by amplification of the 3´ untranslated region of exon 2 (5´-CCTTGAGCCTCTCACCCTTTTGTCTC-3´ and 5´-CTCCTATTGTTCCCAACAGTTGCC-3´). A Diphteria Toxin A (*DTA*) gene was used for negative selection. The absence of PCR-generated mutations was confirmed by sequencing. The targeting vector was linearized with *Pme*I and electroporated into C57Bl/6 ES cells. ES cells were selected with 200 µg/ml G418. Homologous recombination events at the 5´and 3´ arms were verified in G418 resistant ES cell clones by PCR and Southern blot analysis. Six independent clones were injected into C57BL/6J blastocysts. Chimeric mice were crossed with a C56BL/6 Cre-deleter mouse strain (GenOway) to allow germline excision of the *loxP*-flanked region thus generating heterozygous constitutive SMUG1 knockout mice. The novel deletion allele (*Smug1*^{tm1Hln}) is here referred to as *Smug1*$^{-/-}$.

Genomic DNA was digested with *PciI,* blotted onto a nylon membrane and hybridized with an external 432 bp probe (ext 3´ probe) located downstream of the short homology arm of the targeting vector. The expected fragment sizes of the wild type and the recombined Cre-excised loci are 9.3 kb and 2.1 kb, respectively. The non-excised allele would give a fragment of 6.3 kb. Pre-hybridization and hybridization were performed at 65 °C for 18 h in hybridization solution (4 × SSC, 1% SDS, 0.5% skimmed milk, 20 mM EDTA, 100 µg/ml herring sperm DNA) followed by washing twice in 3 × SSC, 1% SDS at 65 °C for 15 min and twice in 0.5 × SSC, 1% SDS at 65 °C for 15 min. Bands were visualised after 3 days exposure to BioMax MS films with BioMax intensifying screens. Primers used to generate the external 3′ probe: 5′-CTCATCTGTCTCTTTAATGGTTGGTTGGATG-3′ and 5′-AGCTGGCTAGGGTCACTGTGGAGGTAT-3′.

Mice were genotyped by multiplex PCR. The primers for the *Smug1* alleles were: 5´-GGATGAGGGTTCAGCCAGACCTACA-3´ (forward WT), 5´-

ACTGCGAATATGACTTCAGACATCCCG-3´ (reverse WT), SMUG KO 5´-TGACAGGGTCACATGTCGTACATAA-3´ (forward KO) and SMUG KO 5´-ACTGCGAATATGACTTCAGACATCC3´ (reverse KO). The primers for the *Ung* alleles were: 5´-CACGGACCTAATCAAGCTCACG-3´ (forward WT), 5´-GGCCCACCCTGACAAATCCCC3´ (reverse WT/KO) and 5´-CTTGGGTGGAGAGGCTATTC-3´ (forward KO). AccuPrime *Pfx* Supermix (Invitrogen) was used for the PCR of both *Smug1* and *Ung*. The PCR program for *Smug1* was: 1 cycle of 95 °C for 5 min, 35 cycles of 95 °C for 15 s, 58 °C for 30 s and 68 °C for 1 min and then 1 cycle of 68 °C for 7 min. The PCR program for *Ung* was equal to that of *Smug1* except for the annealing temperature, which was 59 °C. The PCR products were run on 2% agarose gels. The *Smug1* PCR was expected to give a wild type band of 271 bp and a knockout band of 232 bp. The *Ung* PCR gave a wild type band of 550 bp and a knockout band of 850 bp.

Phenotypic assessment of *Smug1*$^{-/-}$ mice

Smug1$^{-/-}$ mice were born at Mendelian ratios and were fertile. Phenotypic assessment of the *Smug1*^{tm1Hln} knockout mouse model was performed according to the modified SHIRPA protocol (EMPRESS, eumorphia.org) at PhenoPro (http://www.phenopro.fr/). Briefly, 10 male wild type and 10 male *Smug1*$^{-/-}$ mice aged 6 weeks old were housed 1 to 4 per cage and fed standard chow diet (D04, Safe) *ad libitum*. Phenotypic testing started at 11 weeks after 3 weeks acclimation in the phenotypic area. Starting from 13 weeks, mice were sent for dysmorphology screening to assess morphological abnormalities in their general physical appearance (weight, length, and dysmorphology, including tail kinks, shape of ears, eyes, head, teeth, limbs, number and shape of digit, irregularities and variation in coat colour, hair distribution and development, irregularities in the genitals). Blood was collected from 14-week-old mice by retro-orbital puncture under isoflurane anaesthesia for biochemistry and hematologic analysis. A complete blood cell count was performed in the Advia 120 workstation. Body composition and bone mineral content were evaluated by X-ray analysis.

Western blot analysis of SMUG1

From the Norwegian mouse cohorts, muscle tissue (10-25 mg) from WT, *Smug1*$^{+/-}$ and *Smug1*$^{-/-}$ mice were homogenised in CK14 homogenisation tubes containing 400 μl T-PER tissue protein extraction reagent (Thermo Scientific) containing 1x protease inhibitor cocktail, cOmplete Mini EDTA-free (Roche) by bead beating. The lysates were recovered as for the genomic DNA isolation (Material and methods). The lysates were centrifuged at 4 °C at maximum speed for 30 min. The supernatants were recovered and the protein concentrations were determined on a DropSense 16 'Touch & Go' reader (Trinean). The extracts were snap-frozen in liquid nitrogen and stored at -80 °C until further use. 75 µg muscle tissue extract extracts were run on a 4–15% Mini-PROTEAN TGX gel and transferred to a nitrocellulose membrane (Invitrogen). One membrane was blocked in 1x PBS pH 7.4, 0.05% tween-20 (PBS-T), 5% milk for 1 h at room temperature and incubated overnight at 4 °C in monoclonal rabbit anti-human SMUG1 antibody (Abcam) diluted 1:1000 in PBS-T. Another membrane was incubated in rabbit anti-GAPDH antibody (Cell Signaling) diluted 1:1000 in PBS-T. The membranes were washed three times for 10 min in PBS-T and then incubated for 2 h at room

temperature in donkey anti-rabbit HRP (Santa Cruz) diluted 1:5000 in PBS-T. The membranes were washed as above before it was developed with SuperSignal West Pico Chemiluminescent kit (Thermo Scientific) according to the instructions from the manufacturer and visualised using a LAS3000 imager (Fujifilm).

Gene expression

From the Norwegian mouse cohorts, RNA from different tissues (kidney, spleen, heart, brain, muscle and liver) from WT, *Smug1*$^{+/-}$, *Smug1*$^{-/-}$ *and* Ung$^{-/-}$Smug1$^{-/-}$ mice were isolated using the mirVana miRNA isolation kit (Ambion). A piece of tissue was added to each CK14 homogenisation tube containing 600 µl ice-cold lysis buffer. The tissue was homogenised and the lysate recovered as before. The lysate was processed further according to manufacturer's instructions. The RNA was eluted in 100 µl DEPC treated milli-Q water (Ambion). cDNA was synthesised from 5 µg RNA using SuperScript II Reverse Transcriptase (Invitrogen/Life Technology) and random hexamers according to the instructions following the transcriptase. *Smug1* transcription was measured by quantitative real-time PCR using Fast SYBR Green Master Mix (Applied Biosystems) according to the instructions following the kit. *Smug1* expression was normalised to *Gapdh* expression. 2 µl cDNA was used in quantitative real-time PCR reactions for both *Smug1* and *Gapdh*. Primers for the qRT-PCR were *Smug1* (forward) 5´-TCAAGTCTTCTTCCGGCACT-3´, *Smug1* (reverse) 5´-ACTCCCACTACCAGACGCAC-3´, *Gapdh* (forward) 5´-AACTTTGGCATTGTGGAAGG-3´ and *Gapdh* (reverse) 5´-GGATGCAGGGATGATGTTCT-3´. Pre-designed QuantiTect primers were used for detecting the mRNA expression levels of *Neil1*, *Nthl1*, *Tdg1* and *Mbd4* (Qiagen). *Gapdh* and *Gusb* were used as housekeeping genes.

Supplementary Figure S1

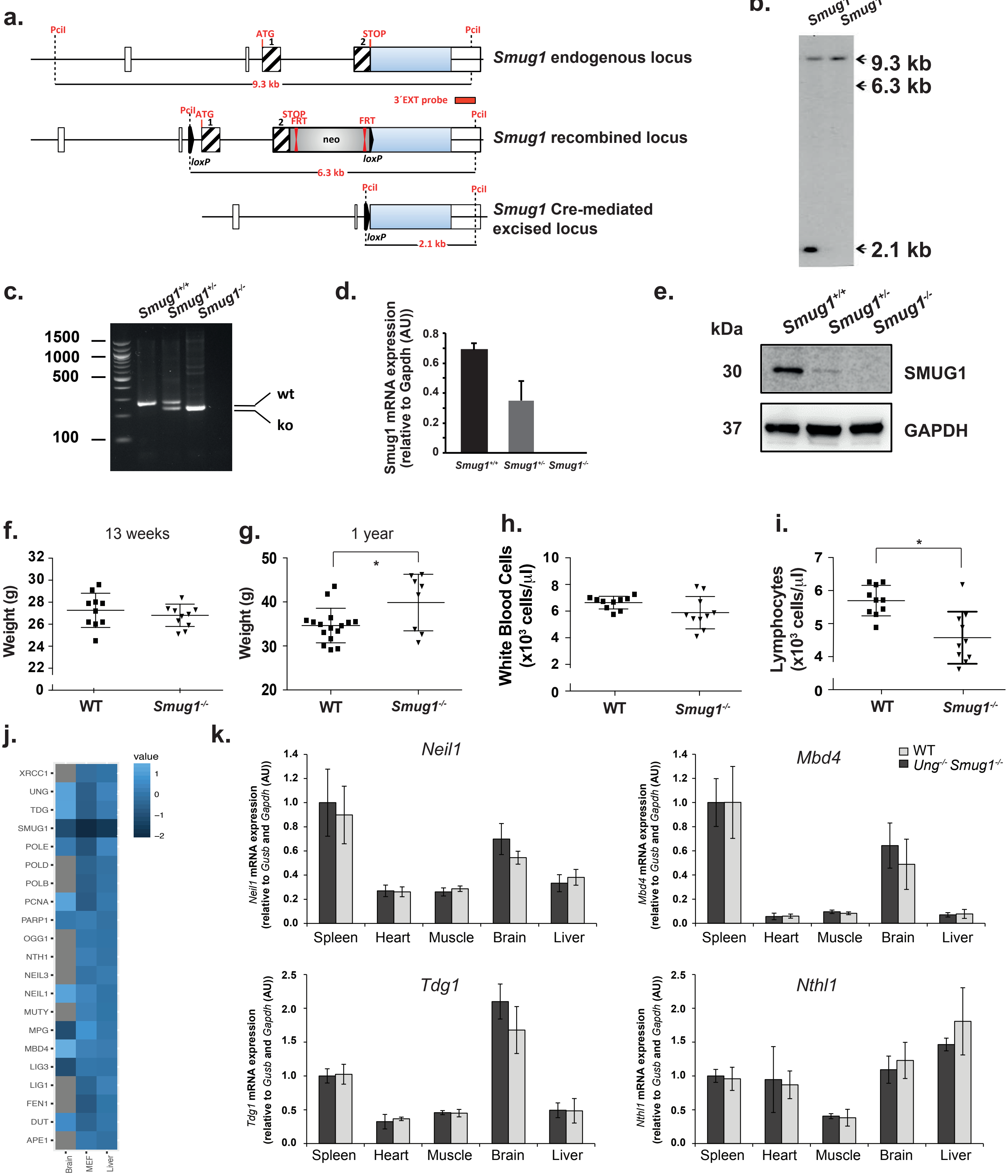

Supplemenraty Figure S1. Generation and characterisation of *Smug1-/-* mice. (a) Gene targeting strategy: endogenous locus (top), two coding exons (striped boxes), 3´UTR (blue), and two predicted upstream exons (white). Recombined locus (middle), with floxed Neomycine selection marker (grey), *LoxP* sites, and the 3´external probe (red). Locus after Cre-mediated excision with PciI sites indicated (bottom). (b) Southern blot confirming Cre-mediated excision. (c) PCR genotyping with bands of expected sizes. (d) qRT-PCR of *Smug1* mRNA expression given as mean of three indep experiments +/- SD. (e) Western blot probed with anti-SMUG1 antibodies. Full blot as shown in the Supplementary information file. (f-i) Phenotping of wild type (squares) and *Smug1-/-* male mice (triangles) showed normal body weight at 13-weeks. (g) The average weight of 6 wild type and 8 *Smug1-/-* male mice sacrificed at 1 year of age showed that *Smug1-/-* mice were somewhat heavier: 39.9 ± 6.4 g compared to the wild type weight of 34.6 ± 3.9 g (p = 0.02). (h) No major change was observed in blood-cell counts although there was a slight reduction in the mean number of white blood cells, (i) attributed to a reduction in lymphocytes numbers (5.70 ± 0.15 vs 4.57 ± 0.25, p = 0.0011) in the *Smug1-/-* mice. The numbers of white blood cells and lymphocytes are represented as mean +/- SEM of ten 13-week old male mice of each genotype. Significant differences were assessed by unpaired Student´s t-test. (j) RNA sequencing heatmap showing Log2-fold changes of the indicated genes in *Smug1-/-* vs wild type tissues. None of the genes were significantly differentially regulated. (k) qRT-PCR analyses of *Neil1*, *Mbd4*, *Tdg1* and *Nthl1* mRNA expression in *Ung-/-Smug1-/-* mice showing no difference in the tissues analysed. Both *Gusb* and *Gapdh* were used as housekeeping genes. Data are shown as mean±SD from three mice of each genotype.

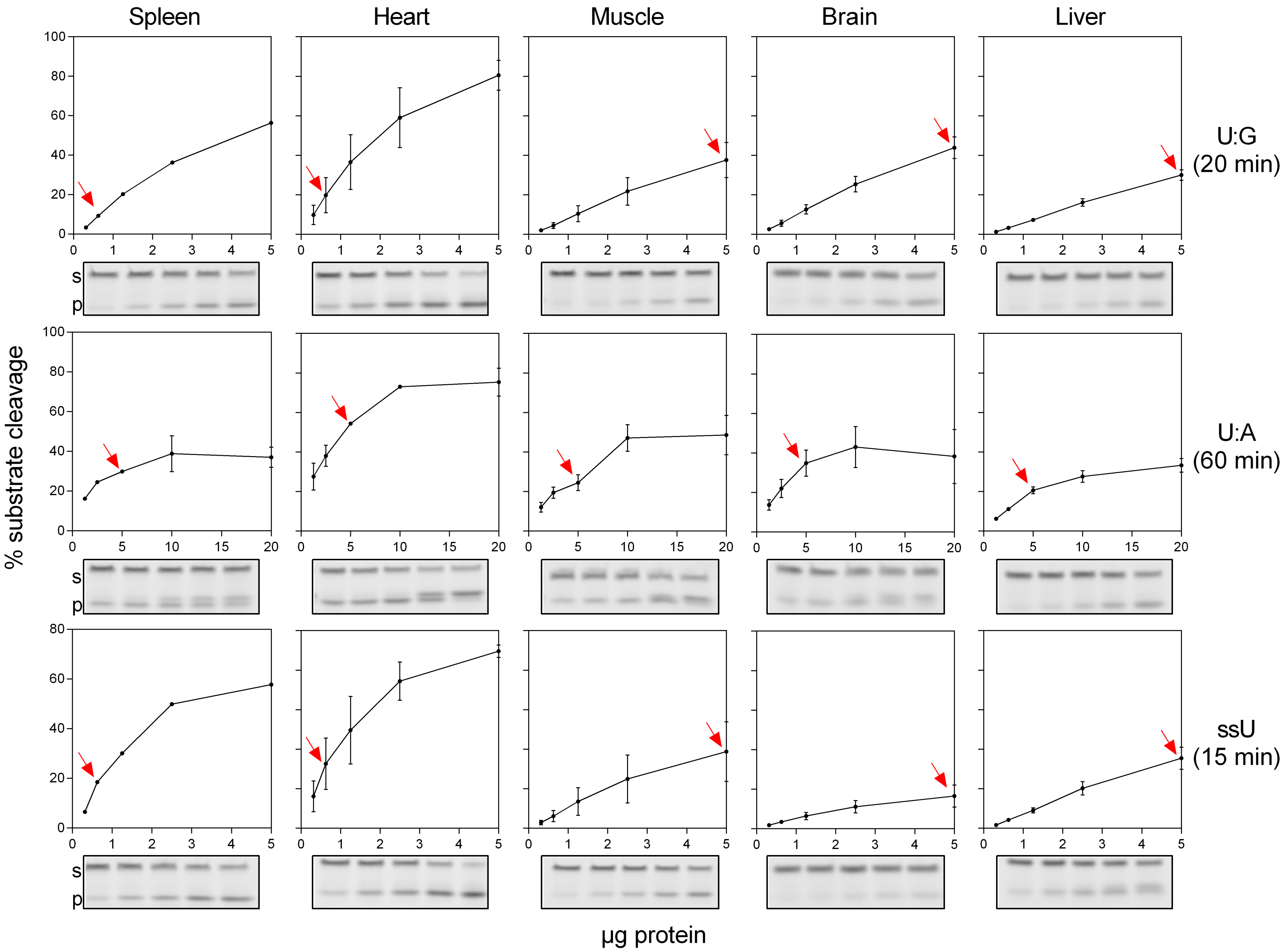

Supplementary Figure S2. Uracil excision activity assay optimisation. Uracil excision activity was measured using different cell extract protein concentration from WT mice. Incubation times depending on substrate: 15, 20, and 60 min for ssU, U:G, and U:A substrates, respectively. Red arrows indicate which concentration was used in subsequent experiments. Error bars indicate SD of two biological replicates.

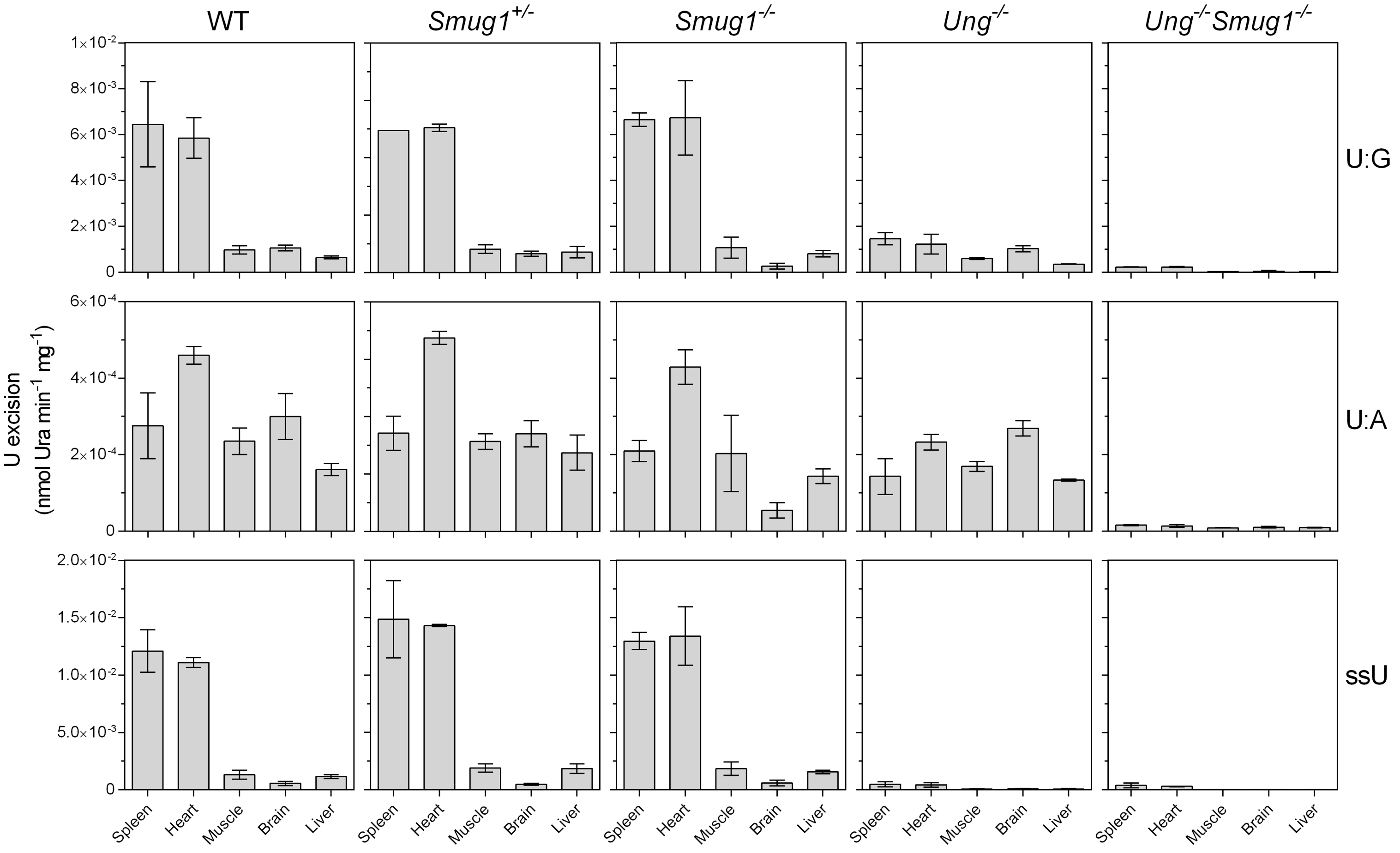

Supplementary Figure S3. *Ung$^{-/-}$Smug1$^{-/-}$* mice lose all uracil excision activity and accumulate a large amount of genomic uracil. Uracil excision activity measured in U:G (top row), U:A (middle row) and single-stranded (lower panel) oligonucleotides. SMUG1 knockout has no measurable effect on U-excision activity in the presence of UNG except for in brain extracts on U:G and U:A contexts. U:G and U:A excision activities is decreased in *Ung$^{-/-}$* mice in all organ extracts except the brain and completely ablated in all organs in *Ung$^{-/-}$Smug1$^{-/-}$* mice. Single-stranded uracil excision activities are ablated both in *Ung$^{-/-}$* extracts and in *Ung$^{-/-}$Smug1$^{-/-}$* mice.

Supplementary Figure S4

a.

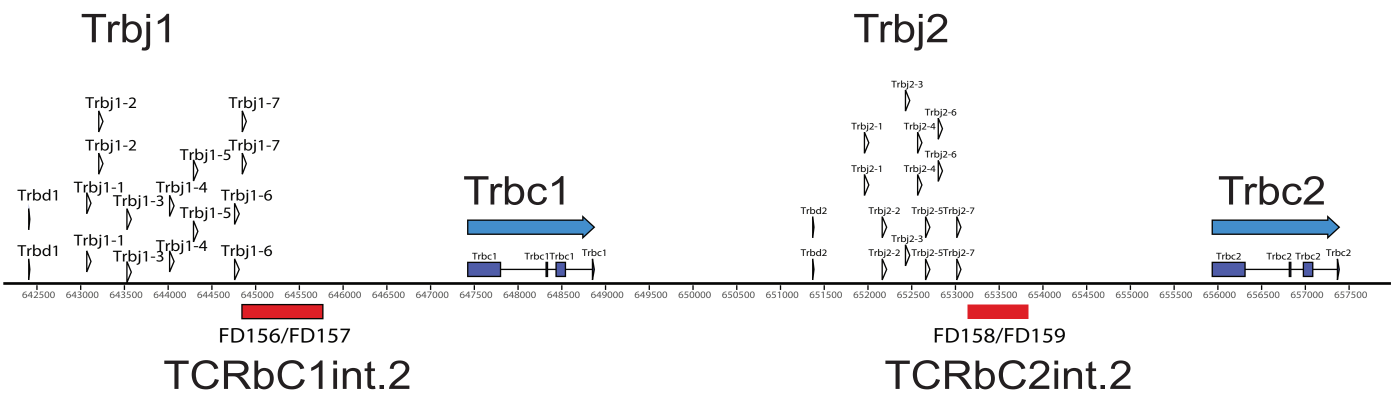

b.

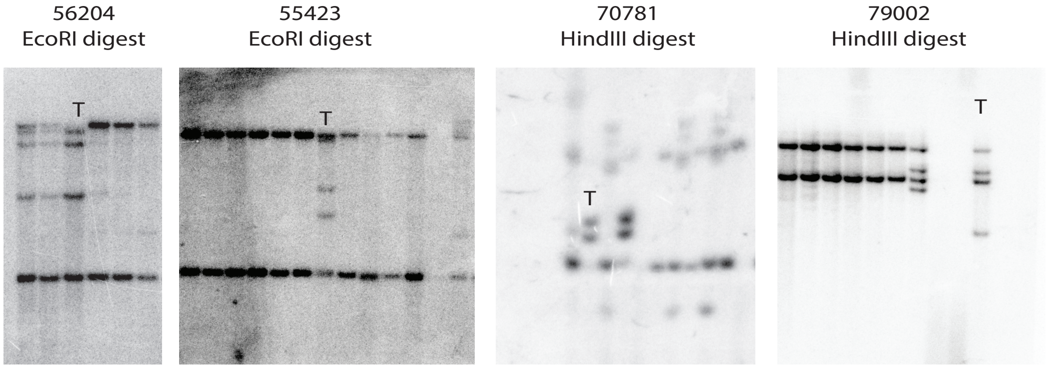

Supplementary Figure S4. Characterisation of clonality of lymphomas

(a) Illustration of the T-cell receptor locus with binding sites of probes (red) and the primers used to generate the probes indicated (red). T-cell receptor beta constant (*Trbc*) genes 1 and 2 are shown (blue) with the exon structure indicated below (purple). (b) Southern blot showing screening of mouse tissue for clonality by probing for clonal T-cell receptor rearrangements. The predominant bands represent the germline configuration; T indicates the lane of the sequenced tumour samples for each mouse.

Supplementary Figure S5

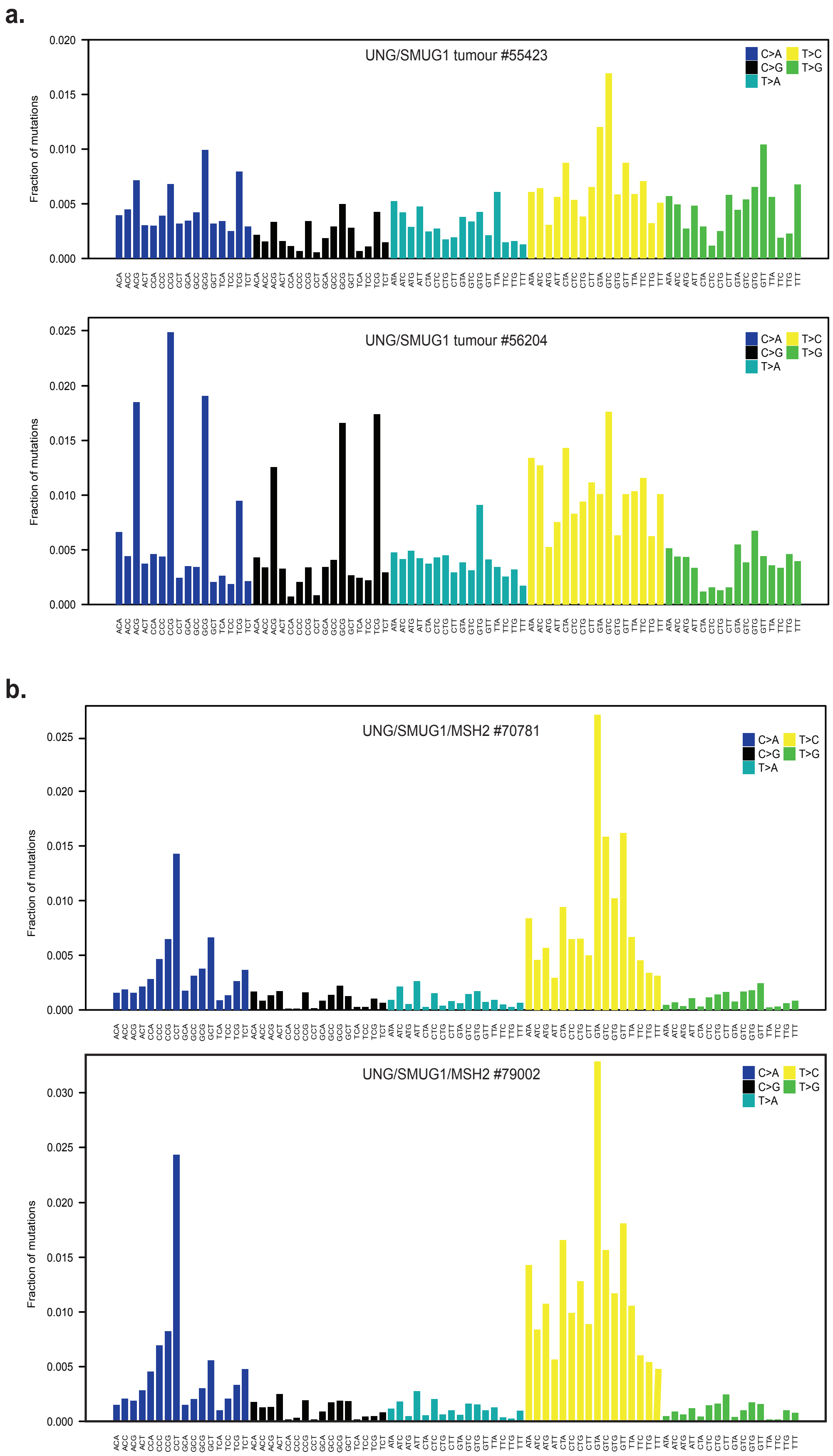

Supplementary Figure S5. Trinucleotide plots of somatic variants in thymic lymphoma
Trinucleotide plots for all variants except C to T transitions summarising the sequences surrounding each variant observed in (a) UNG/SMUG1 double knockout tumours 55423 (top) and 56204 (bottom) panel and (b) UNG/SMUG1/MSH2 triple knockout tumours 70781 (top) and 79002 (bottom) panel.

Supplementary Figure S6

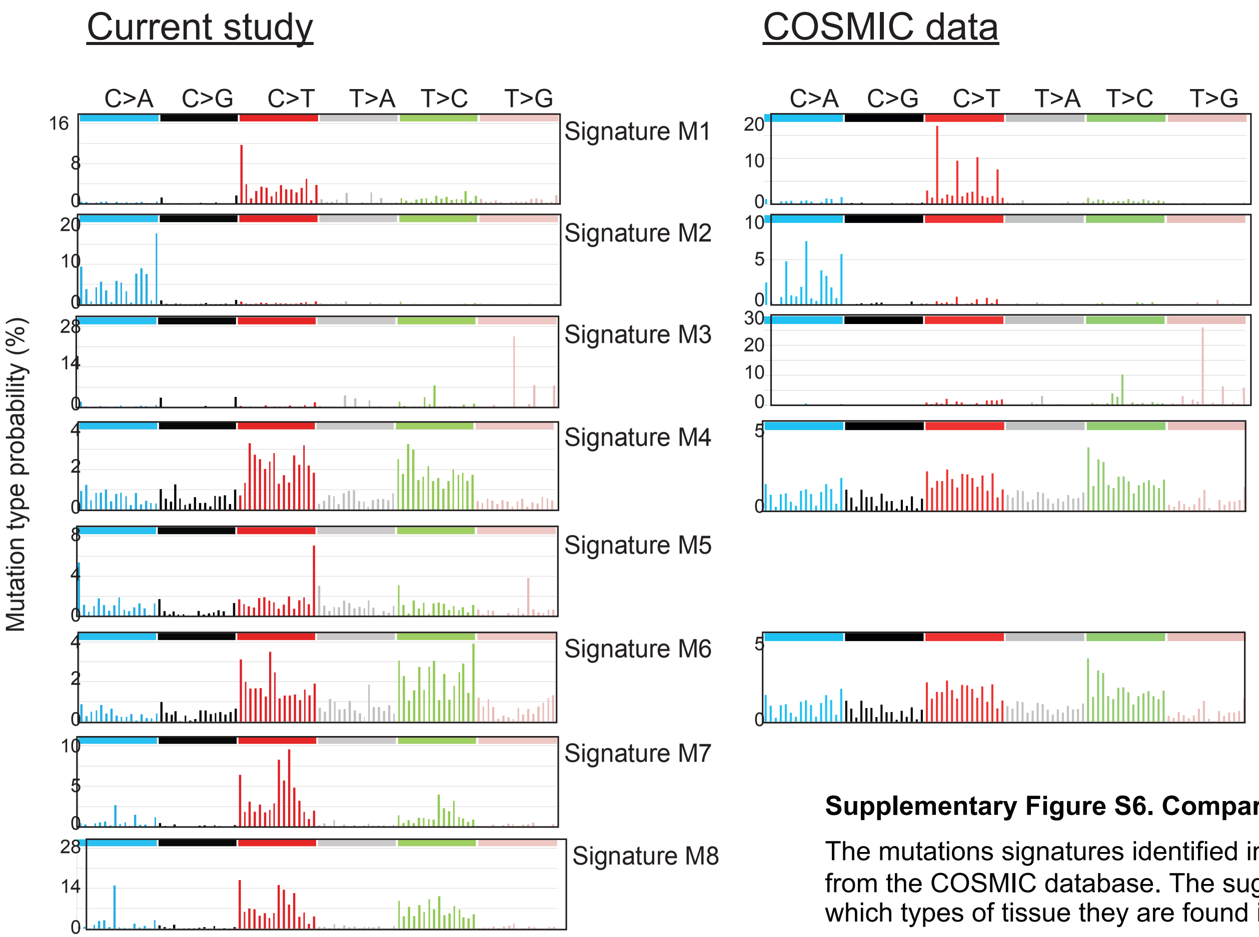

Current study	COSMIC data	Aetiology	Cancer type associations
Signature M1	Signature 1	5-methylcytosine deamination	all cancer types
Signature M2	Signature 18	unknown	neuroblastoma; breast and stomach carcinomas
Signature M3	Signature 17	unknown	oesophagus cancer, breast cancer, liver cancer, lung adenocarcinoma, B-cell lymphoma, stomach cancer, melanoma
Signature M4	Signature 5	unknown	all cancer types
Signature M5			
Signature M6	Signature 5	unknown	all cancer types
Signature M7			
Signature M8			

Supplementary Figure S6. Comparisons of mouse and COSMIC mutation signatures

The mutations signatures identified in our study are shown next to similar mutation signatures from the COSMIC database. The suggested origin of the COSMIC mutation signatures and in which types of tissue they are found is also shown.

Supplementary Table S1. Number* of mutations observed.

Tumour no	C>A	C>G	C>T	T>A	T>C	T>G	Total
55423 (UNG/SMUG1-KO)	670	300	5098	780	1663	1202	9713
56204 (UNG/SMUG1-KO)	1553	1154	6267	2049	5270	1889	18182
70781 (UNG/SMUG1/MSH2-KO)	2557	541	14339	925	7012	900	26274
79002 (UNG/SMUG1/MSH2-KO)	4116	802	17507	1250	12316	1134	37125

- Variants in simple repeats and low complexity regions are excluded

Supplementary Table S2 Sequence contexts* of mutations**

Tumour 55423

C>A + G>T mutations	Observations					Percentages observed				Percentages expected				Diff			
Pos	A	C	G	T	Sum	A	C	G	T	A	C	G	T	A	C	G	T
-3	223	133	140	177	673	33%	20%	21%	26%	29%	21%	21%	29%	4%	-1%	0%	-3%
-2	225	132	122	194	673	33%	20%	18%	29%	29%	21%	21%	29%	4%	-1%	-3%	0%
-1	187	164	146	176	673	28%	24%	22%	26%	29%	21%	21%	29%	-1%	3%	1%	-3%
0	0	673	0	0	673	0%	100%	0%	0%	0%	100%	0%	0%	0%	0%	0%	0%
1	234	174	60	205	673	35%	26%	9%	30%	29%	21%	21%	29%	6%	5%	-12%	1%
2	234	116	122	201	673	35%	17%	18%	30%	29%	21%	21%	29%	6%	-4%	-3%	1%
3	204	120	138	211	673	30%	18%	21%	31%	29%	21%	21%	29%	1%	-3%	0%	2%

C>G + G>C mutations	Observations					Percentages observed				Percentages expected				Diff			
Pos	A	C	G	T	Sum	A	C	G	T	A	C	G	T	A	C	G	T
-3	103	55	60	82	300	34%	18%	20%	27%	29%	21%	21%	29%	5%	-3%	-1%	-2%
-2	76	75	70	79	300	25%	25%	23%	26%	29%	21%	21%	29%	-4%	4%	2%	-3%
-1	90	43	99	68	300	30%	14%	33%	23%	29%	21%	21%	29%	1%	-7%	**12%**	-6%
0	0	300	0	0	300	0%	100%	0%	0%	0%	100%	0%	0%	0%	0%	0%	0%
1	97	70	30	103	300	32%	23%	10%	34%	29%	21%	21%	29%	3%	2%	-11%	5%
2	77	60	89	74	300	26%	20%	30%	25%	29%	21%	21%	29%	-3%	-1%	9%	-4%
3	89	60	57	94	300	30%	20%	19%	31%	29%	21%	21%	29%	1%	-1%	-2%	2%

C>T + G>A mutations	Observations					Percentages observed				Percentages expected				Diff			
Pos	A	C	G	T	Sum	A	C	G	T	A	C	G	T	A	C	G	T
-3	1359	1080	1088	1580	5107	27%	21%	21%	31%	29%	21%	21%	29%	-2%	0%	0%	2%
-2	1488	875	996	1748	5107	29%	17%	20%	34%	29%	21%	21%	29%	0%	-4%	-1%	5%
-1	1775	978	1229	1125	5107	35%	19%	24%	22%	29%	21%	21%	29%	6%	-2%	3%	-7%
0	0	5107	0	0	5107	0%	100%	0%	0%	0%	100%	0%	0%	0%	0%	0%	0%
1	2026	1304	667	1110	5107	40%	26%	13%	22%	29%	21%	21%	29%	**11%**	5%	-8%	-7%
2	1370	1464	902	1371	5107	27%	29%	18%	27%	29%	21%	21%	29%	-2%	8%	-3%	-2%
3	1432	1086	1140	1449	5107	28%	21%	22%	28%	29%	21%	21%	29%	-1%	0%	1%	-1%

Tumour 56204

C>A + G>T mutations	Observations					Percentages observed				Percentages expected				Diff			
Pos	A	C	G	T	Sum	A	C	G	T	A	C	G	T	A	C	G	T
-3	476	272	366	444	1558	31%	17%	23%	28%	29%	21%	21%	29%	2%	-4%	2%	-1%
-2	496	392	298	372	1558	32%	25%	19%	24%	29%	21%	21%	29%	3%	4%	-2%	-5%
-1	550	448	279	281	1558	35%	29%	18%	18%	29%	21%	21%	29%	6%	8%	-3%	-11%
0	0	1558	0	0	1558	0%	100%	0%	0%	0%	100%	0%	0%	0%	0%	0%	0%
1	606	326	279	347	1558	39%	21%	18%	22%	29%	21%	21%	29%	**10%**	0%	-3%	-7%
2	567	264	371	356	1558	36%	17%	24%	23%	29%	21%	21%	29%	7%	-4%	3%	-6%
3	497	322	322	417	1558	32%	21%	21%	27%	29%	21%	21%	29%	3%	0%	0%	-2%

C>G + G>C mutations	Observations					Percentages observed				Percentages expected				Diff			
Pos	A	C	G	T	Sum	A	C	G	T	A	C	G	T	A	C	G	T
-3	341	213	250	356	1160	29%	18%	22%	31%	29%	21%	21%	29%	0%	-3%	1%	2%
-2	268	263	244	385	1160	23%	23%	21%	33%	29%	21%	21%	29%	-6%	2%	0%	4%
-1	397	116	299	348	1160	34%	10%	26%	30%	29%	21%	21%	29%	5%	-11%	5%	1%
0	0	1160	0	0	1160	0%	100%	0%	0%	0%	100%	0%	0%	0%	0%	0%	0%
1	371	272	187	330	1160	32%	23%	16%	28%	29%	21%	21%	29%	3%	2%	-5%	-1%
2	300	213	300	347	1160	26%	18%	26%	30%	29%	21%	21%	29%	-3%	-3%	5%	1%
3	329	211	226	394	1160	28%	18%	19%	34%	29%	21%	21%	29%	-1%	-3%	-2%	5%

C>T + G>A mutations	Observations					Percentages observed				Percentages expected				Diff			
Pos	A	C	G	T	Sum	A	C	G	T	A	C	G	T	A	C	G	T
-3	1783	1292	1403	1811	6289	28%	21%	22%	29%	29%	21%	21%	29%	-1%	0%	1%	0%
-2	1481	1295	1396	2117	6289	24%	21%	22%	34%	29%	21%	21%	29%	-5%	0%	1%	5%
-1	1826	1724	1207	1532	6289	29%	27%	19%	24%	29%	21%	21%	29%	0%	6%	-2%	-5%
0	0	6289	0	0	6289	0%	100%	0%	0%	0%	100%	0%	0%	0%	0%	0%	0%
1	1839	1471	1364	1615	6289	29%	23%	22%	26%	29%	21%	21%	29%	0%	2%	1%	-3%
2	1562	1571	1372	1784	6289	25%	25%	22%	28%	29%	21%	21%	29%	-4%	4%	1%	-1%
3	1734	1423	1381	1751	6289	28%	23%	22%	28%	29%	21%	21%	29%	-1%	2%	1%	-1%

Tumour 70781

C>A + G>T mutations	Observations					Percentages observed				Percentages expected				Diff			
Pos	A	C	G	T	Sum	A	C	G	T	A	C	G	T	A	C	G	T
-3	746	485	611	723	2565	29%	19%	24%	28%	29%	21%	21%	29%	0%	-2%	3%	-1%
-2	679	529	577	780	2565	26%	21%	22%	30%	29%	21%	21%	29%	-3%	0%	1%	1%
-1	313	1278	540	434	2565	12%	50%	21%	17%	29%	21%	21%	29%	-17%	**29%**	0%	-12%
0	0	2565	0	0	2565	0%	100%	0%	0%	0%	100%	0%	0%	0%	0%	0%	0%
1	424	457	100	1584	2565	17%	18%	4%	62%	29%	21%	21%	29%	-12%	-3%	-17%	**33%**
2	686	563	631	685	2565	27%	22%	25%	27%	29%	21%	21%	29%	-2%	1%	4%	-2%
3	484	500	564	1017	2565	19%	19%	22%	40%	29%	21%	21%	29%	-10%	-2%	1%	**11%**

C>G + G>C mutations	Observations					Percentages observed				Percentages expected				Diff			
Pos	A	C	G	T	Sum	A	C	G	T	A	C	G	T	A	C	G	T
-3	226	70	92	156	544	42%	13%	17%	29%	29%	21%	21%	29%	**13%**	-8%	-4%	0%
-2	91	169	159	125	544	17%	31%	29%	23%	29%	21%	21%	29%	-12%	**10%**	**8%**	-6%
-1	255	36	163	90	544	47%	7%	30%	17%	29%	21%	21%	29%	**18%**	-14%	**9%**	-12%
0	0	544	0	0	544	0%	100%	0%	0%	0%	100%	0%	0%	0%	0%	0%	0%
1	182	103	42	217	544	33%	19%	8%	40%	29%	21%	21%	29%	4%	-2%	-13%	**11%**
2	126	77	205	136	544	23%	14%	38%	25%	29%	21%	21%	29%	-6%	-7%	**17%**	-4%
3	190	82	123	149	544	35%	15%	23%	27%	29%	21%	21%	29%	6%	-6%	2%	-2%

C>T + G>A mutations	Observations					Percentages observed				Percentages expected				Diff			
Pos	A	C	G	T	Sum	A	C	G	T	A	C	G	T	A	C	G	T
-3	4792	2880	3310	3383	14365	33%	20%	23%	24%	29%	21%	21%	29%	4%	-1%	2%	-5%
-2	3011	2474	3896	4984	14365	21%	17%	27%	35%	29%	21%	21%	29%	-8%	-4%	6%	6%
-1	3528	2310	6576	1951	14365	25%	16%	46%	14%	29%	21%	21%	29%	-4%	-5%	**25%**	-15%
0	0	14365	0	0	14365	0%	100%	0%	0%	0%	100%	0%	0%	0%	0%	0%	0%
1	5025	2909	3493	2938	14365	35%	20%	24%	20%	29%	21%	21%	29%	6%	-1%	3%	-9%
2	3582	2893	3562	4328	14365	25%	20%	25%	30%	29%	21%	21%	29%	-4%	-1%	4%	1%
3	3721	2603	3577	4464	14365	26%	18%	25%	31%	29%	21%	21%	29%	-3%	-3%	4%	2%

Tumour 79002

**C>A + G>T mutations**	Observations					Percentages observed				Percentages expected				Diff			
Pos	A	C	G	T	Sum	A	C	G	T	A	C	G	T	A	C	G	T
-3	1331	862	857	1085	4135	32%	21%	21%	26%	29%	21%	21%	29%	3%	0%	0%	-3%
-2	1146	1074	791	1124	4135	28%	26%	19%	27%	29%	21%	21%	29%	-1%	5%	-2%	-2%
-1	428	2506	517	684	4135	10%	61%	13%	17%	29%	21%	21%	29%	-19%	**40%**	-8%	-12%
0	0	4135	0	0	4135	0%	100%	0%	0%	0%	100%	0%	0%	0%	0%	0%	0%
1	625	671	138	2701	4135	15%	16%	3%	65%	29%	21%	21%	29%	-14%	-5%	-18%	**36%**
2	1052	953	943	1187	4135	25%	23%	23%	29%	29%	21%	21%	29%	-4%	2%	2%	0%
3	778	876	837	1644	4135	19%	21%	20%	40%	29%	21%	21%	29%	-10%	0%	-1%	**11%**

**C>G + G>C mutations**	Observations					Percentages observed				Percentages expected				Diff			
Pos	A	C	G	T	Sum	A	C	G	T	A	C	G	T	A	C	G	T
-3	340	120	154	193	807	42%	15%	19%	24%	29%	21%	21%	29%	**13%**	-6%	-2%	-5%
-2	160	240	214	193	807	20%	30%	27%	24%	29%	21%	21%	29%	-9%	9%	6%	-5%
-1	384	58	246	119	807	48%	7%	30%	15%	29%	21%	21%	29%	**19%**	-14%	9%	-14%
0	0	807	0	0	807	0%	100%	0%	0%	0%	100%	0%	0%	0%	0%	0%	0%
1	224	177	46	360	807	28%	22%	6%	45%	29%	21%	21%	29%	-1%	1%	-15%	**16%**
2	199	117	315	176	807	25%	14%	39%	22%	29%	21%	21%	29%	-4%	-7%	**18%**	-7%
3	255	126	167	259	807	32%	16%	21%	32%	29%	21%	21%	29%	3%	-5%	0%	3%

**C>T + G>A mutations**	Observations					Percentages observed				Percentages expected				Diff			
Pos	A	C	G	T	Sum	A	C	G	T	A	C	G	T	A	C	G	T
-3	5806	3633	3740	4371	17550	33%	21%	21%	25%	29%	21%	21%	29%	4%	0%	0%	-4%
-2	3766	3306	4409	6069	17550	21%	19%	25%	35%	29%	21%	21%	29%	-8%	-2%	4%	6%
-1	4872	2823	7511	2344	17550	28%	16%	43%	13%	29%	21%	21%	29%	-1%	-5%	**22%**	-16%
0	0	17550	0	0	17550	0%	100%	0%	0%	0%	100%	0%	0%	0%	0%	0%	0%
1	6001	4110	3495	3944	17550	34%	23%	20%	22%	29%	21%	21%	29%	5%	2%	-1%	-7%
2	4091	3797	4315	5347	17550	23%	22%	25%	30%	29%	21%	21%	29%	-6%	1%	4%	1%
3	4292	3471	4150	5637	17550	24%	20%	24%	32%	29%	21%	21%	29%	-5%	-1%	3%	3%

** Simple repeats and low complexity regions masked

* APOBEC consensus

APOBEC1	YC	Y=C/T
ABOBEC3G	CCC	W=A/T
ABOBEC3B	TC	R=A/G
AID	WRCY	

Supplementary Table S3. Inverted mutation analysis*

Tumour no	CA+GT	CG+GC	CT+GA	TA+AT	TC+AG	TG+AC	Total
55423	341	192	859	406	499	221	2518
56204	372	88	509	424	380	262	2035
70781	368	175	763	302	457	249	2314
79002	408	156	777	291	479	270	2381

In percent of total:

Tumour no	CA+GT	CG+GC	CT+GA	TA+AT	TC+AG	TG+AC	Total
55423	14%	8%	34%	16%	20%	9%	100%
56204	18%	4%	25%	21%	19%	13%	100%
70781	16%	8%	33%	13%	20%	11%	100%
79002	17%	7%	33%	12%	20%	11%	100%

* Simple repeats and low complexity sequences have been masked
This table is similar to supplementary table S1, but with normal and tumour swapped.

Full Western blot cropped in Supplementary Figure S1e.

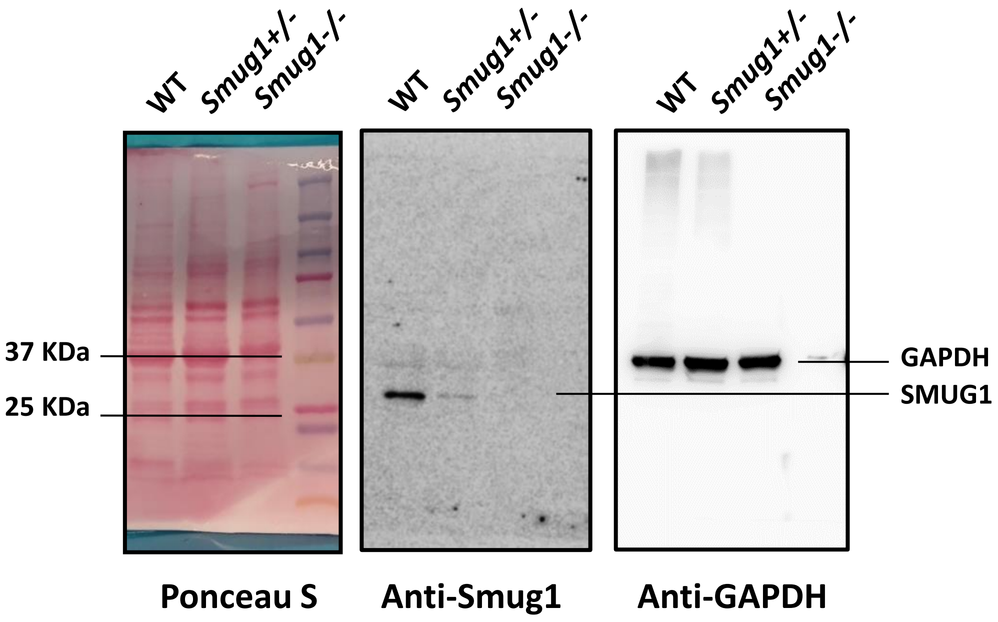

Murine SMUG1 protein expression. Western blotting for SMUG1 (center) using wild type (WT), *Smug1 +/-* and *Smug1-/-* mouse muscle tissue confirmed the respective genotypes. Ponceau S staining (left) and GAPDH immunoblotting (right) confirmed equal protein amount used for each sample. Kaleidoscope® ladder (Biorad) confirmed the expected molecular weights for both SMUG1 and GAPDH.
